# Insecticidal Triterpenes in Meliaceae: Plant Species, Molecules and Activities: Part Ⅰ (*Aphanamixis*-*Chukrasia*)

**DOI:** 10.3390/ijms222413262

**Published:** 2021-12-09

**Authors:** Meihong Lin, Sifan Yang, Jiguang Huang, Lijuan Zhou

**Affiliations:** 1 Key Laboratory of Natural Pesticides and Chemical Biology, Ministry of Education, South China Agricultural University, Guangzhou 510642, China; 24628@noposion.com; 2 Organic Agriculture, Wageningen University and Research, 6708 PB Wageningen, Gelderland, The Netherlands; sifan.yang@wur.nl

**Keywords:** Meliaceae, triterpenoid molecules, insecticidal activities

## Abstract

Plant-originated triterpenes are important insecticidal molecules. The research on insecticidal activity of molecules from Meliaceae plants has always received attention due to the molecules from this family showing a variety of insecticidal activities with diverse mechanisms of action. In this paper, we discuss 102 triterpenoid molecules with insecticidal activity of plants of eight genera (*Aglaia*, *Aphanamixis*, *Azadirachta*, *Cabralea*, *Carapa*, *Cedrela*, *Chisocheton*, and *Chukrasia*) in Meliaceae. In total, 19 insecticidal plant species are presented. Among these species, *Azadirachta indica* A. Juss is the most well-known insecticidal plant and azadirachtin is the active molecule most widely recognized and highly effective botanical insecticide. However, it is noteworthy that six species from *Cedrela* were reported to show insecticidal activity and deserve future study. In this paper, a total of 102 insecticidal molecules are summarized, including 96 nortriterpenes, 4 tetracyclic triterpenes, and 2 pentacyclic triterpenes. Results showed antifeedant activity, growth inhibition activity, poisonous activity, or other activities. Among them, 43 molecules from 15 plant species showed antifeedant activity against 16 insect species, 49 molecules from 14 plant species exhibited poisonous activity on 10 insect species, and 19 molecules from 11 plant species possessed growth regulatory activity on 12 insect species. Among these molecules, azadirachtins were found to be the most successful botanical insecticides. Still, other molecules possessed more than one type of obvious activity, including 7-deacetylgedunin, salannin, gedunin, azadirone, salannol, azadiradione, and methyl angolensate. Most of these molecules are only in the primary stage of study activity; their mechanism of action and structure–activity relationship warrant further study.

## 1. Introduction

Pesticides provide tremendous benefit to modern agriculture. It is well known that the increase of crop yields largely depends on synthetic pesticides. However, it is also recognized that synthetic pesticides have some negative impacts and the indiscriminate application of synthetic pesticides has resulted in contamination of water, soil, air, and crop products, etc. The persistent use of pesticides has also led to serious resistance and resurgence of insect pests [1]. The current consensus asserts that the development of new pesticides should be based on sustainable development, environmental protection, and ecological balance. In order to achieve sustainable development, many scientists have undertaken the search for low toxicity, low residue and environmentally friendly biopesticides, among which botanical pesticides are an important part. Botanical insecticides are attracting global attention as new tools to kill or suppress insect pest populations. Generally, natural products are particularly attractive as templates because of their structural diversity. They can be used directly and have been used as models for the development of several successful insecticides that introduce new mechanisms of action, which are greatly needed to overcome the acquired resistance to synthetic insecticide in agricultural production. Therefore, active chemicals isolated from plants are of considerable significance [2].

The Meliaceae family has 50 genera, including more than 550 species, which are evergreen or deciduous trees or shrubs and are mainly distributed in the tropics and subtropics. These plants are known to be rich sources of limonoids. Until now, various insecticidal active ingredients have been discovered in Meliaceae plants. Numerous studies have demonstrated that the great insecticidal potential of Meliaceae plants has been mainly due to triterpenoids. Many of these triterpenoids have shown contact poison, stomach poison, antifeedant, or growth inhibition activities on various important agricultural insects [3,4,5]. 

This review is an extensive coverage of naturally occurring insecticidal triterpenoids in eight genera (*Aglaia*, *Aphanamixis*, *Azadirachta*, *Cabralea*, *Carapa*, *Cedrela*, *Chisocheton*, and *Chukrasia*) of Meliaceae discovered from 1968 to the present. The insecticidal plant species, insecticidal phytochemicals and their structures, various insecticidal activities, the insecticidal mechanism of action, and the structure–activity relationship (SAR) of the active insecticidal chemicals are summarized. This review thus provides a relatively systemic background on the research of insecticidal triterpenoids from Meliaceae plants and can offer meaningful hints to the development of insecticidal triterpenoids as novel insecticides and promote the application of these molecules in agricultural production.

## 2. Structures of Triterpenes

**Figure 1:**
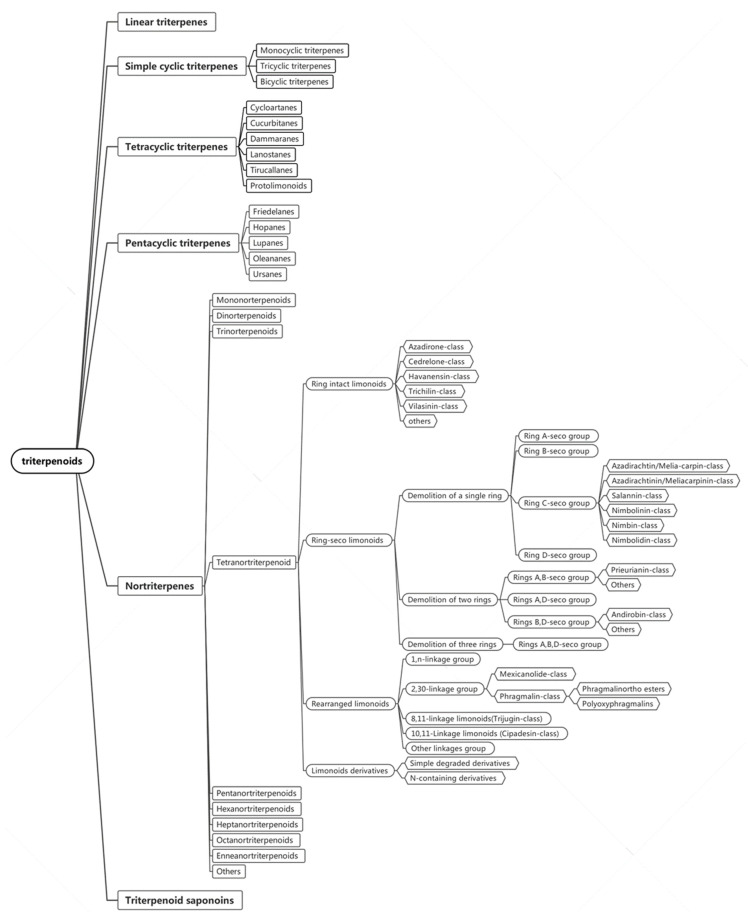
The structural classification of triterpenes.

Triterpenes are terpenoids derived from squalene, usually composed of 30 carbon atoms. The structural classification of triterpenoids is mainly grouped into six groups, including linear triterpenes, simple cyclic triterpenes (monocyclic triterpenes, bicyclic triterpenes, and tricyclic triterpenes), tetracyclic triterpenes, pentacyclic triterpenes, nortriterpenes, and triterpenoid saponins (Figure 1). 

Tetracyclic triterpenes are mainly divided into five groups, including cycloartanes, cucurbitanes, dammaranes, lanostanes, tirucallanes, and protolimonoids; while pentacyclic triterpenoids are mainly divided into five groups, including friedelanes, hopanes, lupanes, oleananes, and ursanes. Simple cyclic triterpenes are further classified into three groups, including monocyclic triterpenes, bicyclic triterpenes, and tricyclic triterpenes. Additionally, triterpenoid saponoinsare saponins are formed by the linkage of hydroxyl groups at certain positions of triterpenoids with different kinds and quantities of sugars [6]. In particular, nortriterpenes are formed by the rearrangement and degradation of triterpenes. Nortriterpenes mainly include mononorterpenoids, dinorterpenoids, trinorterpenoids, tetranorterpenoids, and polynorterpenoids; among them, tetranortriterpenoids are generally found to show obvious insecticidal activities. Specifically, the skeleton of the Meliaceae plant is composed of 26 carbons with the loss of 4 carbons, therefore, they are also called tetranortriterpenoids. 

Tetranortriterpenoids are well-known insecticidal limonoids formed by the loss of the four terminal carbons of the side chain in the apolipoprotein or apolipoane skeleton, and then cyclized to form a 17β-furan ring. The basic skeleton of limonoids undergoes oxidative rearrangement to form various types of limonoids. It is mainly divided into ring intact limonoids, ring-seco limonoids, rearranged limonoids, and limonoids derivatives [7]. 

Among them, ring intact limonoids are mainly classified into five types, including azadirones, cedrelones, havanensins, trichilins, and vilasinins. Particularly, azadirone limonoids are characteristic of 3-oxo-Δ^1,2^ and C-7 oxygenation, while the cedrelone limonoids are 5,6-enol-7-one derivatives. For havanensin limonoids, generally, there exist oxygenic substituents at C-1, C-3, and C-7, and the degree of oxidation of C-28 varies from methyl to carboxyl. In addition, most of the trichilin limonoids contain the C-19/29 lactol bridge and the 14,15-epoxide moieties, while the vilasinin limonoids have the characteristics of a 6α,28-ether bridge [8]. 

Ring-seco limonoids are mainly divided into demolition of a single ring (ring A-seco group, ring B-seco group, ring C-seco group, and ring D-seco group), demolition of two rings (rings A,B-seco group, rings A,D-seco group, and rings B,D-seco group), and demolition of three rings (rings A,B,D-seco group). In particular, the ring C-seco group, which belongs to the group of demolition of a single ring, can be further divided into five classes (azadirachtin/melia-carpin-class, azadirachtinin/meliacarpinin-class, salannin-class, nimbolinin-class, nimbin-class, and nimbolidin-class) [9], while the rings of the A,B-seco group, belonging to the group of demolition of two rings, can be further divided into prieurianin-class and others. In the prieurianin-class, aphanamixoid-type belong to its structural classification [10]. Similarly, rings B,D-seco group also can be further grouped into the andirobin-class and others.

Rearranged limonoids include 1,n-linkage group, 2,30-linkage group, 8,11-linkage group (namely, trijugin-class), 10,11-linkage group (namely, cipadesin-class), and other linkages groups. Among them, 2,30-linkage groups include mexicanolides and phragmalins, and phragmalins can be further divided intophragmalinorthoesters and polyoxyphragmalins.

In addition, limonoid derivatives contain seven types, which are pentanortriterpenoids, hexanortriterpenoids, heptanortriterpenoids, octanortriterpenoids, enneanortriterpenoids, N-containing derivatives, and simple degraded derivatives [9].

## 3. Plant Species and Their Insecticidal Chemicals 

**Figure 2:**
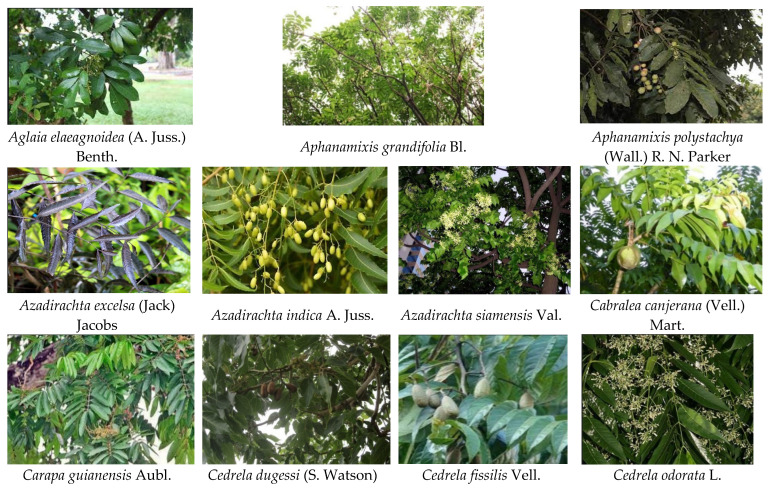

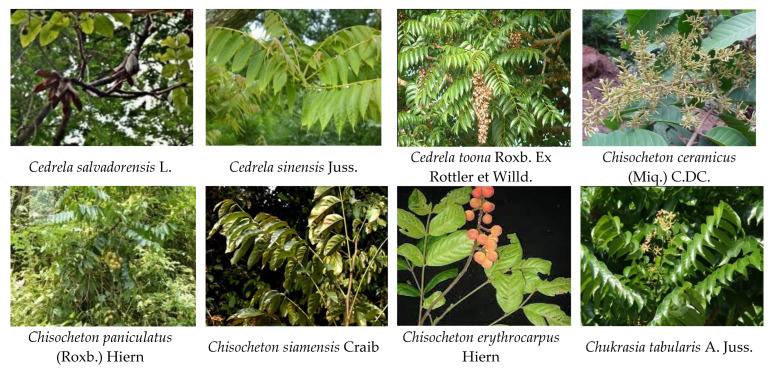
The 19 insecticidal plant species from genera *Aglaia*, *Aphanamixis*, *Azadirachta*, *Carapa*, *Cedrela*, *Cabralea*, *Chisocheton*, and *Chukrasia* in Meliaceae.

A total of 19 insecticidal plant species from eight genera (*Aglaia, Aphanamixis, Azadirachta, Cabralea, Carapa, Cedrela, Chisocheton*, and *Chukrasia*) in Meliaceae are reported here to show insecticidal activities (Table 1 and Figure 2). In these species, *Azadirachta indica* A. Juss was the most well-known insecticidal plant and azadirachtin was the active molecule most widely recognized and highly effective botanical insecticide [10,11,12,13,14,15,16]. However, it is noteworthy that six species from *Cedrela* were reported to show insecticidal activity, deeming them deserving of further study.

In total, 102 insecticidal chemicals were found to be active from the 19 aforementioned plant species. They were active on 29 insect species (*Aedes aegypti* (L.), *Aedes albopictus* Skuse, *Anopheles gambiae* Giles, *Anopheles stephensi* Liston, *Atta sexdens rubropilosa* Forel, *Culex quinquefasciatus* Say, *Diabrotica balteata* Le Conte, *Epilachna paenulata* Germar, *Epilachna varivestis* Mulsant, *Helicoverpa armigera* (Hübner), *Heliothis virescens* (Fabricius), *Heliothis zea* (Boddie), *Leptinotarsa decemlineata* (Say), *Locusta migratoria* (L.), *Musca domestica* L., *Ostrinia nubilalis* (Hübner), *Pectinophora gossypiella* (Saund.), *Peridroma saucia* (Hübner), *Phyllotreta striolata* (Fabricius), *Pieris brassicae* (L.), *Pieris rapae* (L.), *Plutella xylostella* (L.), *Reticulitermes speratus* Kollbe, *Rhodnius prolixus* Stål, *Schistocerca gregaria* Forskål, *Sitobion avenae* (Fabricius), *Spodoptera frugiperda* Smith, *Spodoptera littoralis* (Boisduval), and *Spodoptera litura* (F.)). Generally, these plant-derived chemicals showed good antifeedant, growth inhibition activity, poisonous activity as well as other activities [9,17,18,19,20,21,22,23,24,25,26,27,28,29,30]. 

In sum, 43 chemicals isolated from 15 plant species (*Aphanamixis polystachya* (Wall.) R. Parker, *Azadirachta excelsa* (Jack) Jacobs, *A. indica* A. Juss, *Azadirachta siamensis* Val., *Cabralea canjerana* (Vell.) Mart, *Cabralea eichleriana* DC., *Carapa guianensis* Aubl., *Cedrela dugessi* (S. W atson), *Cedrela fissilis* Vell., *Cedrela odorata* L., *Cedrela salvadorensis* L., *Cedrela sinensis* Juss., *Chisocheton paniculatus* Hiern., *Chisocheton siamensis* Craib, and *Chukrasia tabularis* A. Juss.) showed antifeedant activity against 16 insect species (*E. paenulata*, *E. varivestis*, *H. armigera*, *L. decemlineata*, *L. migratoria*, *O. nubilalis*, *P. saucia*, *P. striolata*, *P. brassicae*, *P. rapae*, *P. xylostella*, *R. speratus*, *R. prolixus*, *S. gregaria*, *S. littoralis*, and *S. litura*) (Table 2) [9,17,21,22,23,29,31]. In these chemicals, azadirachtin, namely azadirachtin A, was the most active and has been successfully used as a botanical insecticide. Azadirachtin B and L also showed significant activity. Normally, the widely used various neem-based insecticide preparations consisted of not only azadirachtin A but also other similar azadirachtins, such as azadirachtin B and L. Still, the activity of other azadirachtins and some other types of chemicals deserves more attention. For example, epoxyprieurianin showed an obvious antifeedant activity on *H. armigera* (EC_50_ = 3.2 μg/mL, 7 d). Another chemical, 1-tigloyl-3-acetyl-azadirachtol, showed good activity on *E. varivestis*. These chemicals could be developed as antifeedant agents on some specific insects in the future [9,32,33].

Overall, 49 chemicals isolated from 14 plant species (*Aglaia elaeagnoidea* (A. Juss.), *A. polystachya*, *A. excelsa*, *A. indica*, *C. canjerana*, *C. eichleriana*, *C. guianensis*, *C. dugessi*, *C. fissilis*, *C. salvadorensis*, *C. sinensis*, *Chisocheton ceramicus* (Miq.) C.DC., *Chisocheton erythrocarpus* Hiern, and *C. paniculatus*) in Meliaceae exhibited poisonous activity on 10 insect species (*A. aegypti*, *A. albopictus*, *A. gambiae*, *A. stephensi*, *A. sexdens rubropilosa*, *C. quinquefasciatus*, *D. balteata*, *P. xylostella*, *S. frugiperda*, and *S. littoralis*) (Table 3) [9,19,20,25,26,28,43]. Normally, the poisonous activity was not the most important of many plant-derived chemicals. However, azadirachtin did show good poisonous activity against *S. littoralis*. Other chemicals such as azadirachtin O, azadirachtin P, azadirachtin Q, azadirachtin B, azadirachtin L, azadirachtin M, 11α-azadirachtin H, and azadirachtol also showed good poisonous activity on *P. xylostella*, with LD_50_ (24 or 96 h) values ranging from 0.75 to 3.92 μg/g [9,33]. 

As a whole, 19 chemicals isolated from 11 plant species (*A. elaeagnoidea*, *A. excelsa*, *A. indica*, *C. canjerana*, *C. guianensis*, *C. fissilis*, *C. odorata*, *C. salvadorensis*, *Cedrela toona Roxb*., *C. paniculatus*, and *C. siamensis*) in Meliaceae possessed growth regulatory activity on 12 insect species (*A. aegypti*, *H. armigera*, *H. virescens*, *H. zea*, *M. domestica*, *O. nubilalis*, *P. gossypiella*, *P. saucia*, *R. prolixus*, *S. frugiperda*, *S. littoralis*, and *S. litura*) and some locusts (Table 4) [17,22,27,30,31,40,43]. Among these chemicals, azadirachtin was the most effective insect growth regulatory agent showing good activity on *H. armigera*, *R. prolixus*, *H. zea*, *H. virescens*, *S. frugiperda*, *P. gossypiella*, *S. litura*, and *S. littoralis*, with EC_50_ or ED_50_ values (7 or 10 d) ranging from 0.11 to 0.70 μg/mL [9,29,30,48,50].

The following sections describe the insecticidal plant species, the corresponding insecticidal chemicals, and their activities in detail.

### 3.1. Aglaia

In the *Aglaia* genus, two species, including *A. elaeagnoidea* and *A. odorata*, have been reported to show insecticidal activity. Previous phytochemical investigation and bioactivity studies on the *Aglaia* genus have shown the main chemical group of this genus to be rocaglamide derivatives (flavaglines) [53]. However, triterpenoids were also the main insecticidal active constituents in this genus.

6α-acetoxygedunin, belonging to ring D-seco limonoids, was isolated from *A. elaeagnoidea* and could reduce the growth of the European corn borer *O. nubilalis* at 50 μg/mL [17,51]. *A. odorata* has been reported to show insecticidal activity on the cotton leafworm *S. littoralis* [54,55]. However, most of the reported compounds with insecticidal activity extracted from this species were rocaglaol derivatives. In addition, some triterpenoids, such as eleganoside A and odoratanone A, have also been reported to be extracted from *A. odorata*, but their insecticidal activities have not been described [56,57,58].

### 3.2. Aphanamixis

*Aphanamixis* is a rich source of limonoids [59,60,61]. In this genus, *A. polystachya* and *A. grandifolia* have been reported to show insecticidal activity (Table 1). 

A total of 17 tetranortriterpenoids were reported to show insecticidal activities. In detail, the 17 tetranortriterpenoids contained 13 rings A,B-seco-type limonoids (prieurianin, epoxyprieurianin, zaphaprinin I, zaphaprinin R, aphapolynin A, aphapolynin C, aphapolynin F, aphapolynin D, dregenana-1, aphanamixoid A, aphanamixoid C, aphanamixoid F, and aphanamixoid G) [18,31,62] and 4 ring A-seco type chemicals (aphanalide E, aphanalide F, aphanalide G, and aphanalide H) [19,34]. 

#### 3.2.1. Rings A,B-seco Limonoids

In this group, 13 chemicals have been reported to show insecticidal activity and they were prieurianin, epoxyprieurianin, zaphaprinin I, zaphaprinin R, aphapolynin A, aphapolynin C, aphapolynin F, aphapolynin D, dregenana-1, aphanamixoid A, aphanamixoid C, aphanamixoid F, and aphanamixoid G). These chemicals were isolated from *A. polystachya* [17,31,62,63].

In these chemicals, prieurianin and epoxyprieurianin exhibited antifeedant activity against the cotton bollworm, *H. armigera* and the EC_50_ values were 18.8 μg/mL and 3.2 μg/mL, respectively, after 7 d [34]. Further study has shown that prieurianin-type limonoids, zaphaprinin I, showed strong insecticidal activities against the aphid *S. avenae*, with a mortality score of 99, which was the same with the positive control thiamethoxam. Both Zaphaprinin I and Zaphaprinin R showed strong insecticidal activities against the diamondback moth/cabbage moth, *P. xylostella* and both mortalities were scored as 99, which was the same with the positive control thiamethoxam [63].

Aphapolynin A has been found to cause a mortality score of 66 against the diamondback moth *P. xylostella* in a leaf-disk assay at 500 μg/mL. Mortality was assessed relative to untreated control wells, with wells showing significant levels of mortality scored as 99, and wells without significant mortality scored as 0 [19,64]. Similarly, aphapolynin C, aphapolynin D, aphapolynin F, and dregenana-1 were found to possess obvious insecticidal activity against the banded cucumber beetle, *D. balteata* in a leaf-disk assay at 500 μg/mL [19,34,65].

Aphanamixoids are a novel class of limonoids derived from prieurianin-type limonoids. Aphanamixoid A, aphanamixoid C (highly oxidized tetra-uridine), aphanamixoid F, and aphanamixoid G all affected the feeding activity of the cotton bollworm, *H. armigera*. The EC_50_ values of these compounds (24 h) were 0.015, 0.017, 0.008, and 0.012 μmol/cm^2^, respectively [18,31,66].

#### 3.2.2. Ring A-seco Limonoids

Aphanalide E, aphanalide F, aphanalide G, and aphanalide H were found to cause mortalities scored as 33–99 against the banded cucumber beetle *D. balteata* in a leaf-disk assay at 500 μg/mL at 5–9 days. Mortality was assessed relative to untreated control wells, with wells showing significant levels of mortality scored as 99, and wells without significant mortality scored as 0 [19,64].

### 3.3. Azadirachta

In this genus, three species, *A. indica*, *A. excels*, and *A. siamensis* were reported to show insecticidal activity with triterpenoids.

A total of 36 tetranortriterpenoids (21 ring-seco limonoids and 15 ring intact limonoids), 7 pentanortriterpenoids (11α-azadirachtin H, azadirachtin I, azadirachtin L, azadirachtin M, azadirachtin P, nimbinene, and nimbandiol), 2 octanortriterpenoids (desfurano-6α-hydroxyazadiradione and desfuranoazadiradione), and 2 protolimonoids (meliantriol and odoratone) were reported to show insecticidal activities [9,22,23,24,26,27,32,33,35,39].

Specifically, the 21 ring-seco limonoids were mainly the demolition of a single ring, consisting of 18 ring C-seco limonoinds (12 azadirachtin/meliacarpin-class chemicals, 5 salannins, and 1 nimbin-class chemical), and 3 ring D-seco limonoids (gedunin, 7-deacetylgedunin and 6β-hydroxygedunin). Further, the 12 azadirachtin/meliacarpin-class chemicals were azadirachtin A, azadirachtin B, azadirachtin D, azadirachtin E, azadirachtin F, azadirachtin G, azadirachtin K, azadirachtin N, azadirachtin O, azadirachtin Q, azadirachtol, and 1-tigloyl-3-acetylazadirachtol. The 5 salannins were salannin, 3-deacetylsalannin, salannol, 3-O-acetyl salannol, and nimbolide. Additionally, the only nimbin-class chemical was 6-deacetylnimbin. As far as the 15 ring intact limonoids were concerned, they were 13 azadirones (nimocinolide, isonimocinolide, azadirone, 7-deacetylazadiradione, 7-deacetyl-17β-hydroxyazadiradione, 17β-hydroxyazadiradione, 23-O-methylnimocinolide, 7-O-deacetyl-23-O-methyl-7α-O-senecioylnimocinolide, nimocinol, 6α-O-acetyl-7- deacetylnimocinol, 22,23-dihydronimocinol, epoxyazadiradione, and azadiradione), and 2 other ring intact limonoids (azadiraindin A and meliatetraolenone) [9,22,23,24,26,27,32,33,35,39].

#### 3.3.1. Ring C-seco Chemicals

In this group, 18 chemicals were reported to show insecticidal activity: azadirachtin A, azadirachtin B, azadirachtin D, azadirachtin E, azadirachtin F, azadirachtin G, azadirachtin K, azadirachtin N, azadirachtin O, azadirachtin Q, azadirachtol, 1-tigloyl-3-acetylazadirachtol, salannin, 3-deacetylsalannin, 3-O-acetyl salannol, salannol, 6-deacetylnimbin, and nimbolide [9,22,23,32,33,39].

Among these chemicals, azadirachtins were the most widely used botanical insecticides originating from *A. indica* [67,68,69] and *A. excels* [20,32,36]. Presently, azadirachtins contain 15 analogs, 10 of which (azadirachtin A, B, D, E, F, G, K, N, O, and Q) belong to azadirachtin/meliacarpin-class chemicals and 5 of which (11α-azadirachtin H, I, L, M, and P) belong to pentanortriterpenoids [70]. As far as the insecticidal activity was concerned, Azadirachtin A, B, L, O, P, Q, and M gained wide attention [9,33,39].

Normally, azadirachtin is referred to as azadirachtin A [9]. Azadirachtin A has a broad control spectrum. It was reported that azadirachtin A possessed strong insecticidal activities against more than 400 insect species in Lepidoptera, Hymenoptera, Coleoptera, and so on. Azadirachtin A has shown various activities, including antifeeding, growth inhibition, repellent, stomach poisoning, and sterilizing [10,11,12,13,14,15,16]. Particularly, antifeeding and growth inhibition activities were the most remarkable [71,72,73]. Azadirachtins and neem-based formulations included liquid type, pellet type, alginate-biosorbent, and so on [74,75]. Of note, there are more than 2000 references focusing on azadirachtins and several reviews on azadirachtins. Further information can be referred to in the papers by Mordue (1993), Kraus (1993), Ley (1994), and Devakumar (2009) [21,41,76,77,78,79,80,81,82,83,84,85,86,87].

3-O-acetyl salannol, salannol, and salannin have shown growth inhibitory activity on the cotton bollworm *H. armigera* and the tobacco cutworm *S. litura*. After 7 days, the EC_50_ values of them on *H. armigera* were 64.2, 79.7, and 86.5 μg/mL, respectively. Similarly, the EC_50_ values of them on *S. litura* were 65.6, 77.4, and 87.7 μg/mL, respectively [22]. Meanwhile, these three chemicals together with 3-deacetylsalannin were also reported to show antifeedant activity on insects. In a choice leaf disc bioassay, after 7 days, 3-O-acetyl salannol, salannol, and salannin reduced feeding by 50% in *S. litura* at 2.0, 2.3, and 2.8 µg/cm^2^, respectively [22]. Salannin also showed antifeedant activity on the lower subterranean termite *R. speratus* and the PC_95_ value was 203.3 μg/disc after 30 d. In contrast, 3-deacetylsalannin showed a weak antifeedant activity on *R. speratus* and the PC_95_ value was 1373.1 μg/disc after 30 d [23].

In this group, another chemical nimbolide, isolated from *A. indica* and *A. excels,* could inhibit the feeding of the Mexican bean beetle, *E. varivestis*. The EC_50_ value was 90 μg/mL [9,32,88]. Nimbin-class chemical 6-deacetylnimbin showed antifeedant activity on the lower subterranean termite *R. speratus*. The PC_95_ value was 1581.2 μg/disc after 30 days [23].

#### 3.3.2. Ring D-seco Chemicals

In this group, three chemicals were reported to show insecticidal activity and they were gedunin, 7-deacetylgedunin, and 6β-hydroxygedunin.

Gedunin showed antifeedant activity on the lower subterranean termite *R. speratus* (PC_95_, 113.7 μg/disc) and growth inhibitory activity on the cotton bollworm *H. armigera* (EC_50_, 50.8 μg/mL) and the tobacco cutworm *S. litura* (EC_50_, 40.4 μg/mL). In contrast, the derivative of gedunin, 7-deacetylgedunin, was reported to show a weaker antifeedant activity on the lower subterranean termite *R. speratus* (PC_95_, 218.4 μg/disc) after 30 days. However, in artificial diet bioassays, 6β-hydroxygedunin showed better growth inhibitory activity on the cotton bollworm *H. armigera* (EC_50_, 24.2 μg/mL, 7 d) and the tobacco cutworm *S. litura* (EC_50_, 21.5 μg/mL, 7 d). This efficacy was higher in comparison to gedunin, the EC_50_ (7 d) of which on *H. armigera* and *S. litura* were 50.8 and 40.4 μg/mL, respectively [23,35].

#### 3.3.3. Rings Intact Limonoids: Azadirones, Azadiraindin A and Meliatetraolenone

As mentioned above, there were 13 azadirones: azadirone, azadiradione, epoxyazadiradione, 7-deacetylazadiradione, 17β-hydroxyazadiradione, and 7-deacetyl-17β- hydroxyazadiradione, nimocinol, 22,23-dihydronimocinol, 6α-O-acetyl-7-deacetylnimocinol, nimocinolide, isonimocinolide, 23-O-methylnimocinolide, and 7-O-deacetyl-23-O-methyl-7α- O-senecioylnimocinolide.

Azadirone showed antifeedant activity against the Colorado potato beetle *L. decemlineata* with an antifeedant index of 11.6 ± 6.3 (100 μg/mL) (starved for 6 h and feed for 20 h) [37]. Azadiradione and epoxyazadiradione were also reported to show antifeedant activities to some extent against the diamondback moth *P. xylostella* [24]. Further, azadiradione, 7-deacetylazadiradione, and 7-deacetyl-17β-hydroxyazadiradione were isolated from the seeds of *A. indica* and they showed growth inhibitory activity against the tobacco budworm *H. virescens* and the EC_50_ values were 560, 1600, and 240 μg/mL, respectively. Similarly, 17β-hydroxyazadiradione also showed antifeedant activity and the PC_95_ value at the lower subterranean termite *R. speratus* was 235.6 μg/disc after 30 days [23].

Nimocinol, 6α-O-acetyl-7-deacetylnimocinol, 23-O-methylnimocinolide, and 7-O-deacetyl-23-O-methyl- 7α-O-senecioylnimocinolide poseessed insecticidal activity on the mosquito *A. aegypti*. The LC_50_ (24 h) values of them were 21.0, 83.0, 53.0, and 2.14 μg/mL, respectively [25,45].

Nimocinolide, isonimocinolide and 22,23-dihydronimocinol were also isolated from the fresh leaves of *A. indica* [26,27]. Nimocinolide and isonimocinolide affected the fecundity of the housefly *M. domestica* at 100–500 μg/mL and showed mutagenic properties in the mosquito *A. aegypti*. In contrast, 22,23-dihydronimocinol showed poisonous activity on the mosquito *A. stephensi* and the LC_50_ value was 60 μg/mL after 24 h [26].

Additionally, the other ring intact limonoids, azadiraindin A and meliatetraolenone, were reported to show insecticidal activity. Azadiraindin A showed antifeedant activities against the diamondback moth *P. xylostella*. The antifeedant rate was 28% at 2000 μg/mL after 48 h [24]. Meliatetraolenone, isolated from the leaves of *A. indica*, showed insecticidal activities against the mosquito *A. stephensi* and the LC_50_ value was 16 μg/mL after 24 h [44].

#### 3.3.4. Pentanortriterpenoids

In this group, seven chemicals have been reported to show insecticidal activity and they were 11α-azadirachtin H, azadirachtin I, azadirachtin L, azadirachtin M, azadirachtin P, nimbinene, and nimbandiol. There were five kinds of azadirachtin analogs (11α-azadirachtin H, I, L, M, and P) that belonged to pentanortriterpenoids. 11α-azadirachtin H, azadirachtin L, azadirachtin M, and azadirachtin P, which were reported to have insecticidal activities, were isolated from the seed kernels of *A. excelsa*. The LD_50_ values (24 h) of these derivatives against the diamondback moth *P. xylostella* were 5.75, 10.27, 8.46, and 2.19 μg/g, respectively [33].

Nimbinene exhibited growth inhibitory activity on insects and the EC_50_ values of nimbinene on the cotton bollworm *H. armigera* and the tobacco cutworm *S. litura* were 391.4 and 404.5 μg/mL, respectively after 7 days [35]. Further, nimbandiol were found to show antifeedant activity on the lower subterranean termite *R. speratus* and the PC_95_ values was 254.4 μg/disc after 30 days [23].

#### 3.3.5. Octanortriterpenoids

Desfurano-6α-hydroxyazadiradione, isolated from fresh leaves of *A. indica*, showed insecticidal activity on the mosquito *A. stephensi* and the LC_50_ value was 43 μg/mL after 24 h [39]. Comparatively, desfuranoazadiradione showed relatively weak antifeedant activity on the diamondback moth *P. xylostella* to some extent as demonstrated by the low mortality rate (39.6% after 48 h) at a high concentration (2000 μg/mL) [24].

#### 3.3.6. Protolimonoids

Odoratone, isolated from the leaves of *A. indica*, showed insecticidal activities against the mosquito *A. stephensi* and the LC_50_ value was 154 μg/mL after 24 h [44]. Another protolimonoid isolated from this plant was meliantriol, found to be a feeding inhibitor preventing locust chewing [52].

### 3.4. Cabralea

In this genus, *C. canjerana* has been reported to show insecticidal activity.

From *C. canjerana*, 2 tetracyclic triterpenes (cabraleadiol and ocotillone) and 2 tetranortriterpenoids were isolated and shown to have insecticidal activity [41,48]. Particularly, cabraleadiol and ocotillone belonged to dammaranes, while 3-β-deacetylfissinolide was one of mexicanolides. Furthermore, the 2 tetranortriterpenoids consisted of 1 ring B, D-seco limonoid (methyl angolensate), and 1 rearranged limonoid (3-β-deacetylfissinolide) [40,48]. Other known compounds such as gedunin and 7-deacetoxy-7-oxogedunin (belonging to ring D-seco limonoids) were also contained in these plants [89].

Ocotillone and methyl angolensate showed antifeedant activity on the tobacco cutworm *S. litura*. At 1μg/cm^2^, the PFI (percentage feeding index) values (24 h) of the two chemicals were 44.5 and 65.3, respectively [40,41,90,91,92,93]. Additionally, they also showed insecticidal activity at 50 mg/kg with a mortality rate of 40% for the larva of the fall armyworm *S. frugiperda* after 7 d [48,94]. Cabraleadiol and 3-β-deacetylfissinolide affected the larval development on *S. frugiperda*. At 50 mg/kg, when treated by the method of semi-artificial diet, the larval phase was extended by 1.2 d [48].

### 3.5. Carapa

In this genus, until now, only *C. guianensis* has been reported to show insecticidal activity [95,96].

From this species, a ring D-seco limonoid β-photogedunin and a ring intact limonoid 17β-hydroxyazadiradione were reported to show insecticidal activity [17,97]. Particularly, 17β-hydroxyazadiradione belong to azadirones. Other known compounds such as gedunin and 7-deacetoxy-7-oxogedunin were also contained in these plants [28].

At 50 mg/kg, β-photogedunin, when treated by the method of semi-artificial diet, reduced the weight of pupa the fall armyworm *S. frugiperda*. Meanwhile, the mortalities caused by β-photogedunin on the larval and pupal of *S. frugiperda* were 53.3% and 20.0% (7 d), respectively. In contrast, gedunin at 50 mg/kg caused a mortality of 63.3% to the larval *S. frugiperda* after 7 d [48,93]. 17β-hydroxyazadiradione showed antifeedant activity on the lower subterranean termite *R. speratus* with a PC_95_ (95% protective concentrations, μg/disc) value (30 d) of 235.6 μg/disc [23,98,99].

### 3.6. Cedrela

In the genus *Cedrela,* six species, *C. dugessi, C. fissilis*, *C. odorata, C. salvadorensis*, *C. sinensis*, and *C. toona* have been reported to show insecticidal activity [100,101].

From these species, 25 tetranortriterpenoids (1 ring intact limonoid (cedrelone), 15 ring-seco limonoids, 9 rearranged limonoids) and 2 pentacyclic triterpenes (oleanolic acid and oleanonic acid) were reported to show insecticidal activity. Specifically, the 15 ring-seco limonoids included 10 ring D-seco type chemicals (gedunin, photogedunin epimer mixture, 6α-acetoxy-gedunin, 7-deacetylgedunin, photoacetic acid acetate mixture, 7-deacetoxy-7-oxogedunin, photogeduninepimeric mixture, photogeduninepimeric acetate mixture, photogedunin, and 1,2-dihydro-3β-hydroxy-7-deacetoxy-7-oxogedunin) [28,47,49,102], 4 rings A, D-seco type chemicals (11β,19-diacetoxy-l-deacetyl-l-epidihydronomilin, 11β-acetoxyobacunyl acetate, 11β-acetoxyobacunol and odoralide) [29], and 1 rings B,D-seco type chemical cedrelanolide I [44]. The nine rearranged limonoids consisted of eight mexicanolides (swietenolide, swietemahonolide, 3β-acetoxycarapin, 8β,14α-dihydroswietenolide, 3β,6-dihydroxydihydrocarapin, 3β-hydroxyindoline, xyloccensin K, cedrodorin) and cipadesin B, a chemical belonging to 10,11-linkage limonoids [29,103]. In contrast, the above-mentioned mexicanolides belong to the 2,30-linkage group.

#### 3.6.1. The Ring Intact Limonoid: Cedrelone

Cedrelone showed no antifeedant effect. However, cedrelone could affect the development and reproduction of the variegated cutworm *P. saucia.* After 9 days of feeding, the EC_50_ value of growth inhibition of cedrelone on *P. saucia* was found to be 53.1 μg/mL. By injection to the 6th instar of *P. saucia*, cedrelone inhibited growth, delayed development, and resulted in considerable larval mortality [43,50,104,105].

#### 3.6.2. Ring D-seco Limonoids

Gedunin, photogedunin epimer mixture, and photoacetic acid acetate mixture have shown insecticidal activity. The LC_50_ values (7 d) of these compounds against the fall armyworm *S. frugiperda* were shown to be 39, 10, and 8 μg/mL, respectively [47,49,97,105]. Photogedunin, 6α-acetoxy-gedunin, 7-deacetylgedunin, 7-deacetoxy-7-oxogedunin, and 1,2-dihydro-3β-hydroxy-7-deacetoxy-7-oxogeduni possessed insecticidal activity on the leaf-cutting ant, *A. sexdens rubropilosa*. At 100 μg/mL, the S_50_ values (S_50_—survival average 50% (S_50_)/d) of these chemicals on *A. sexdens rubropilosa* varied from 8 to 11 d [28,106]. When treated with photogeduninepimeric acetate mixture at 10 μg/mL, the survival rate of the fall armyworm *S. frugiperda* was 50%. However, the photogeduninepimeric mixture showed a higher activity, as shown by the 17% survival rate of *S. frugiperda* when treated at 10.0 μg/mL after 24 h [49].

#### 3.6.3. Rings A,D-seco Limonoids and Rings B,D-seco Limonoids

At 1000 μg/mL, 11β,19-diacetoxy-l-deacetyl-l-epidihydronomilin, 11β-acetoxyobacunyl acetate, 11β-acetoxyobacunol, and odoralide showed antifeedant activity on the cotton leafworm *S. littoralis* [29]. At 50 μg/mL, cedrelanolide I exhibited a significant weight reduction on the European corn borer *O. nubilalis* [51].

#### 3.6.4. The Rearranged Limonoids

8β,14α-dihydroswietenolide showed antifeedant activity on the cotton leafworm *S. littoralis*, which was active at 500 μg/mL. Swietemahonolide and 3β-acetoxycarapin possessed insecticidal activity on the leaf-cutting ant *A. sexdens rubropilosa*. At 100 μg/mL, both S_50_ values of swietemahonolide and 3β-acetoxycarapin were 8 d [103]. Swietenolide, xyloccensin K, cedrodorin, and 3β,6-dihydroxydihydrocarapinand 3β-hydroxydihydrocarapin showed antifeedant activity on the cotton leafworm *S. littoralis* at 1000 μg/mL [29].

As for the 10,11-linkage limonoid cipadesin B, it was reported to possess an effect on *A. sexdens rubropilosa*. At 100 μg/mL, the S_50_ values of cipadesin B on *A. sexdens rubropilosa* was 9 d [103].

#### 3.6.5. Pentacyclic Triterpenes

The two pentacyclic triterpenes, oleanolic acid and oleanonic acid, belong to oleanane triterpenes. They were reported to possess an effect on *A. sexdens rubropilosa* and the S_50_ values of oleanolic acid and oleanonic acid at 100 μg/mL on this insect were 6 d and 8 d, respectively [103].

### 3.7. Chisocheton

Four species, *C. ceramicus*, *C. paniculatus*, *C. siamensis*, and *C. erythrocarpus*, have been reported to exhibit insecticidal activity.

From *C. paniculatus*, three ring intact limonoids, azadiradione, 7-deacetylazadiradione (namely, nimbocinol), chisocheton compound F, and 2 mexicanolides (14-deoxy-Δ^14,15^-xyloccensin K, 14-deoxyxyloccensin K), were reported to exhibit insecticidal activity. Particularly, the three chemicals belonged to azadirones. Azadiradione was isolated from the acetone/hexane (1:1) extract of the seeds of *C. siamensis.* Moreover, gedunin was also contained in this plant [38,46,107,108].

Azadiradione showed growth inhibitory activity on the tobacco budworm *H. virescens.* The EC_50_ value (EC_50_ value was the effective concentration of additive necessary to reduce larval growth to 50% of the control values) was 560 μg/mL. In addition, the EC_50_ of its alkaline hydrolysis product, 7-deacetylazadiradione, was 1600 μg/mL [27,30,109]. Chisocheton compound F, isolated from *C.* *paniculatus*, showed antifeedant activity against the large white butterfly *P. brassicae* [38].

Mexicanolides 14-deoxy-Δ^14,15^-xyloccensin K and 14-deoxyxyloccensin K, isolated from *C. ceramicus* and *C. erythrocarpus*, showed larvicidal activity on the mosquitoes *A. aegypti*, *A. albopictus*, and *C. Quinquefasciatus.* After 24 h, the LC_50_ values of 14-deoxy-Δ^14,15^-xyloccensin K on them were 10.2, 12.16, and 16.82 μg/mL, respectively; while the LC_50_ values of 14-deoxyxyloccensin K on them were 3.19, 3.01, and 3.64 μg/mL, respectively [46].

### 3.8. Chukrasia

*C. tabularis* has been reported to show insecticidal activity.

From this species, five rearranged limonoids, belonging to tetranortriterpenoids, were isolated. Specifically, they were phragmalins, which belonged to the 2,30-linkage group of the rearranged limonoids. The five chemicals were tabulalin, tabulalide A, tabulalide B, tabulalide D, and tabulalide E. They all showed antifeedant activity against the third instar larvae of the cotton leafworm *S. littoralis*. Among them, tabulalin and tabulalide D were active at 500 μg/mL. Tabulalides A, B, and E were active at 1000 μg/mL at 2–12 h after the treatment [42,110,111,112,113].

## 4. Structures and Structure–Activity Relationship (SAR) of the Insecticidal Chemicals

### 4.1. Structures of the Insecticidal Chemicals

In total, 102 insecticidal chemicals have been summarized, including 96 nortriterpenes, 4 tetracyclic triterpenes, and 2 pentacyclic triterpenes. The structures of the chemicals are shown in Figure 3, Figure 4, Figure 5, Figure 6, Figure 7, Figure 8, Figure 9, Figure 10, Figure 11, Figure 12, Figure 13, Figure 14, Figure 15, Figure 16, Figure 17, Figure 18, Figure 19, Figure 20 and Figure 21.

The 96 nortriterpenes include 87 tetranortriterpenoids, 7 pentanortriterpenoids, and 2 octanortriterpenoids. Further, the 87 tetranortriterpenoids contain 17 ring intact limonoids, 53 ring-seco limonoids, and 17 rearranged limonoids. Specifically, the 53 ring-seco limonoids include 4 ring A-seco chemicals, 18 ring C-seco limonoids, 12 ring D-seco limonoids, 13 rings A,B-seco limonoids, 4 rings A,D-seco limonoids, and 2 rings B,D-seco limonoids. The 17 rearranged limonoids include 16 2,30-linkage limonoids and one 10,11-linkage limonoid.

### 4.2. Structure–Activity Relationship (SAR) of the Insecticidal Chemicals

Traditional insecticide discovery effectively contributes to the development of new insecticides but is limited by high costs and long cycles. Structure–activity relationship (SAR) methods were introduced to evaluate the activity of compounds virtually, which saves significant costs for determining the activities of the compounds experimentally [114].

An SAR study on the antifeedant effects and developmental delays of three different azadirachtin A derivatives against *E. varivestis* showed that the hydroxy group at C-11 is important for high mortality rates and a single bond between C-22 and C-23 increases the degree of efficiency. An exchange of the large ester group ligands at C-1 and C-3 with hydroxy groups in combination with a single bond between C-22 and C-23 and a hydroxy group at C-11 leads to high feeding activity and a degree of efficiency of about 100% [115]. Interestingly, another study aiming to understand the structure-related bioactivities of the limonoids based on the insect antifeedant and growth-regulating activities of 22 limonoids (both natural and their derivatives) against the tobacco cutworm, *S. litura*, indicated that the C-seco limonoids (azadirachtins A, B, D, H, and I) were the most effective compounds as a group, while the intact limonoids (cedrelone and its derivatives) were the least effective. The cyclohexenone A ring and the α-hydroxy enone group in the B ring appear to be important for antifeedant activity. The presence of a cyclohexenone or 1,2-epoxide in the A ring coupled with an α-hydroxy enone in the B ring correlated well with growth regulatory activity. An acetoxy at C-7 instead of α-hydroxy enone, and perhaps the carbonyl at C-16, increase growth regulatory activity. The absence of 14–15 epoxide may not drastically reduce antifeedant activity and growth regulatory activity [41].

Based on 25 limonoids isolated from the fruits of *A. polystachya*, including seven new prieurianin-type limonoids, aphapolynins C-I, and one new C3-C6 connected aphanamolide-type limonoid aphanamolide B, along with 17 known compounds, a structure–activity analysis revealed that the α,β-unsaturated lactone and 14,15-epoxy moieties were essential for insecticidal activity [19]. Further structure–activity relationship analysis of the aphanamixoids indicated that the olefinic bond, the Δ^2,30^ configuration, and the substituent at C-12 significantly affected the antifeedant potency [18]. Antifeedant effect comparison of prieurianin, prieurianin acetate, epoxyprieurianin, and epoxyprieurianin acetate revealed that, first, epoxy compounds are more efficacious and, second, that acetylation enhances the activity of these rings A,B-seco-type limonoids [34].

A structure–activity study based on 11 molecules (nimbandiol, 17-hydroxyazadiradione, deacetylnimbin, 17-epiazadiradione, deacetylsalannin, azadiradione, nimbin, and deacetylgedunin), gedunin, salannin, and epoxyazadiradione) revealed that the furan ring, αβ-unsaturated ketone, and hydroxyl group each played an important role in determining the antifeedant activity. Specifically, a hydroxyl group at C-7 increased the antifeedant activity of gedunin [23]. Later, a further structure–activity study revealed that a hydroxyl group at C-7 reduced the insect growth inhibitory activity and the antifeedant activity of azadiradione, while a hydroxyl group at C-17 increased the activity of azadiradione and 7-deacetylazadiradione. Compared with 7-deacetylazadiradione, the parent natural product contained hydroxyl groups at both the C-7 and C-17 positions, which might contribute to the activity [27,30,109]. Hydroxyl groups in other groups of limonoids were also found to influence biological activity. For example, acetylation or ketonization of the C-7 or C-l 2 hydroxyl groups in the trichilins rendered them inactive as antifeedants against larvae of the southern armyworm, *S. eridania* (Cramer). On the other hand, deacetylation of the C-1 acetate group in nomilin rendered it inactive as a growth inhibitor against larvae of the fall armyworm and the corn earworm [23,30]. Additionally, comparison of the activities of β-photogedunin and gedunin indicated that oxidation of the furan ring led to a decrease in insecticidal activity [48].

An SAR study of rearranged limonoids was also investigated. By comparision of the antifeedant activity of tabulalin, tabulalide D, tabulalide E, tabulalide A, chukvelutilide I, chukvelutilide N, chukvelutilide J, chukvelutilide K, chukvelutilide L, tabulalide B, chukvelutilides O, and chukvelutilides M on the third instar larvae of the cotton leafworm, *S. littoralis*, it was concluded that acylation of the 30-hydroxy group on the tricyclodecane ring system reduced activity [42,110,111,112,113].

## 5. Insecticidal Mechanism of Action

A study of the insecticidal mechanism of action (MOA) of triterpenoids mainly focused on the MOA of azadirachtin with few MOA studies on other molecules. For example, it was demonstrated that both rings A,B-seco-type limonoids aphapolynin C and aphanalide H inhibited a nicotine response with IC_50_ at 3.13 μg/mL (aphapolynin C) and 1.59 μg/mL (aphanalide H), respectively, and aphanalides H also inhibited a GABA response with IC_50_ at 8.00 μg/mL [19]. Currently, azadirachtin is widely recognized as one of the most promising plant compounds for pest control in organic agriculture and one of the best alternatives to conventional insecticides in IPM programs [71,116]. The MOA study of azadirachtin has been a hot topic. However, even after many years of study, the exact molecular mechanism of action of azadirachtin has yet to be fully understood [117,118]. So far, the principal azadirachtin action on insects could be categorized into four groups: effects on neuro-endocrine activity, effects on reproduction, anti-feedancy, and cellular and molecular effects [116].

The primary antifeeding effect of azadirachtin seems to be mediated by gustatory chemosensillas and linked to inhibition on the rate of firing of sugar-sensitive cells of the gustatory chemoreceptors by activating bitter sensitive gustatory cells [119,120,121]. An internal feedback mechanism called secondary antifeedancy, including a long-term reduction in food intake, and deleterious effects on different insect tissues (muscles, fat body, gut epithelial cells), has also been reported [122,123,124]. In addition, azadirachtin showed an agonistic effect on dopaminergic neurons and can induce aversive taste memory in *Drosophila melanogaster*, and such memory is regulated by dopaminergic signals in the brain resulting in inhibition of the proboscis extension response (PER) [125].

Azadirachtin is an antagonist of 20-hydroxyecdysone (20E) and juvenile hormone (JH), two principal hormones in insects. The major action of azadirachtin has been its effect on hemolymph ecdysteroid and JH titers by inhibition of the secretion of morphogenetic peptide hormone (PTTH) and allatotropins from the corpus cardiacum complex, resulting in the IGD effects such as a failure of adult emergence, reduced pupation, or malformation. Moreover, azadirachtin could influence the activity of ecdysone 20-monooxygenase, which is a cytochrome P450-dependant hydroxylase responsible for the conversion of the steroid hormone ecdysone to its more active metabolite, and 20E. Furthermore, azadirachtin can cause degenerative structural changes in the nuclei in all endocrine glands (prothoracic gland, corpus allatum, and corpus cardiacum) responsible for controlling molting and ecdysis in insects, which would contribute to a generalized disruption of neuroendocrine function [117,122]. It was reported that the inhibition of growth and development in the fruit fly, *D. melanogaster*, after azadirachtin treatment was similar to those caused by disruption of the IIS pathway. In addition, azadirachtin can inhibit the excitatory cholinergic transmission and partly block the calcium channel, and this might interfere with different endocrinological and physiological actions in insects [126].

Owing to the interference of azadirachtin with yolk protein synthesis and or its uptake into oocytes, azadirachtin reduced the fecundity and fertility of several insects [127]. Sterility effects in females due to interference with vitellogenin synthesis and uptake into oocytes were also reported. In males, azadirachtin significantly decreases the number of cysts and the apical nuclei within the cysts in *D. melanogaster*, thereby inhibiting spermiogenesis [128,129,130]. In addition, azadirachtin was found to alter reproductive behavior, mating behavior, and oviposition behavior [128,131].

Additionally, the molecular insecticidal mechanisms of azadirachtin have been investigated and several explanations have been presented. For instance, it was found that azadirachtin could induce apoptosis through caspase-dependent pathways and could also inhibit protein synthesis and release by binding to specific proteins (such as heat-shock protein, hsp 60), affected genes encoding key enzymes such as the gene encoding cytochrome oxidase-related proteins CYP307A1 and CYP314A1, which catalyze the 20-hydroxyecdysone [132], and the gene encoding JH epoxide hydrolase, responsible for JH degradation by hydrolyzing the epoxide of JH [133,134,135].

In sum, recent work has demonstrated the MOA of azadirachtin to be complex and is not yet fully understood. Therefore, continued research is needed to reveal the ultimate MOA.

## 6. Future Outlook

Research on the insecticidal activity of Meliaceae plants has always received considerable attention. Investigations of Meliaceae plants over the past decades have led to some significant achievements.

Azadirachtin is the most successful botanical insecticide among the active compounds extracted from Meliaceae. Accordingly, the progress of the worldwide application of azadirachtin in controlling insect pests is inspiring. The application of azadirachtin can control insects, and at the same time, be safe for non-target arthropods. Such work demonstrates the effectiveness of a phytochemical for sustainable pest control in contrast to any negative effects of synthetic insecticide use.

In addition to azadirachtin, some azadirachtin analogs have also demonstrated strong insecticidal activities. Moreover, some compounds in Meliaceae possess more than one type of favorable activity, such as 7-deacetylgedunin, salannin, gedunin, azadirone, salannol, azadiradione, and methyl angolensate; some of which have multiple activities (poisoning, antifeeding, or growth inhibition). Among them, 7-deacetylgedunin and gedunin can be extracted from many Meliaceae plants. However, they are still in the primary stages of research and further studies on these compounds are needed. Their activities on insects should be systemically evaluated as well as their effects on non-target organisms and the environment. It is expected that 7-deacetylgedunin, gedunin, and so on, could be important molecules for managing insect pests in the near future.

Most of the compounds with obvious activity are only in the primary stages of research, and their mechanism of action and structure–activity relationship warrant further study. Generally, tetranortriterpenoids have complex structures and are difficult to synthesize. Therefore, it is of considerable significance to study the synthesis of tetranortriterpenoids with outstanding activity in Meliaceae.

## Figures and Tables

**Figure 3:**
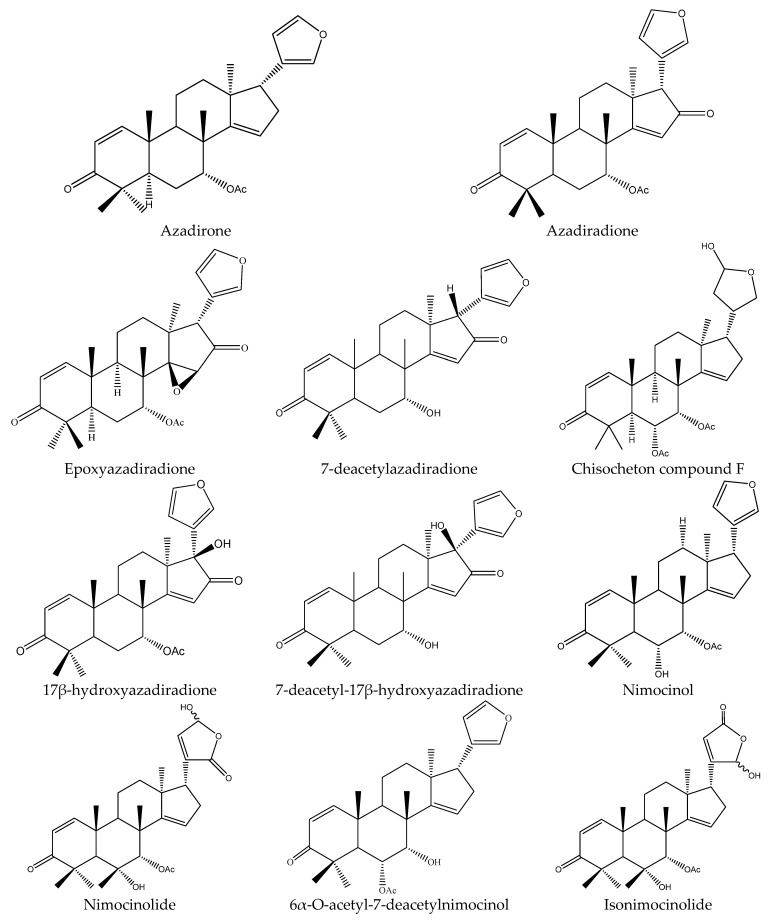

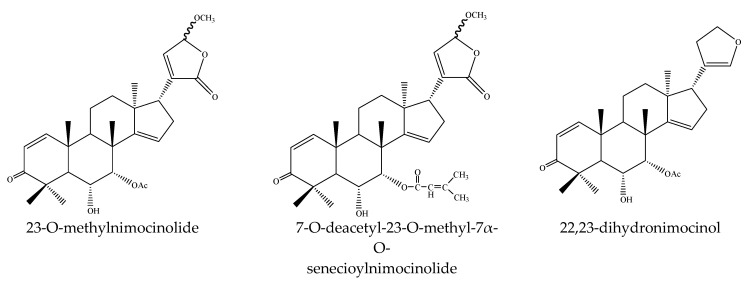
The structures of ring intact limonoids: azadirones.

**Figure 4:**
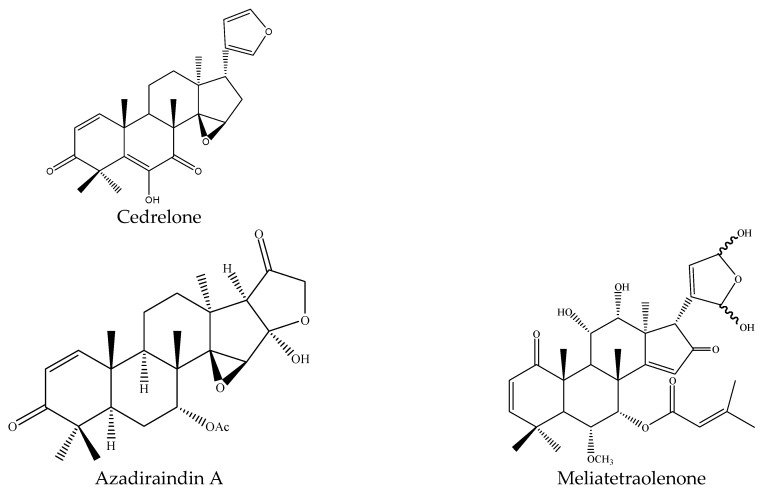
The structure of ring intact limonoids: cedrelone, azadiraindin A, and meliatetraolenone.

**Figure 5:**
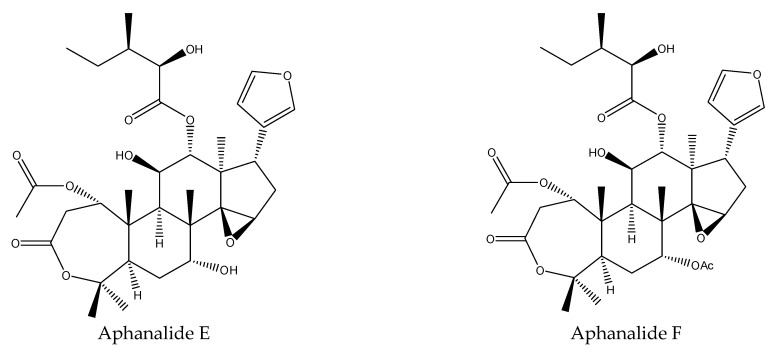

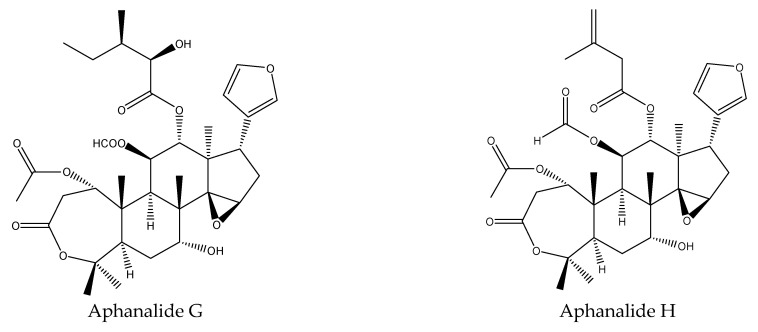
The structures of ring A-seco chemicals.

**Figure 6:**
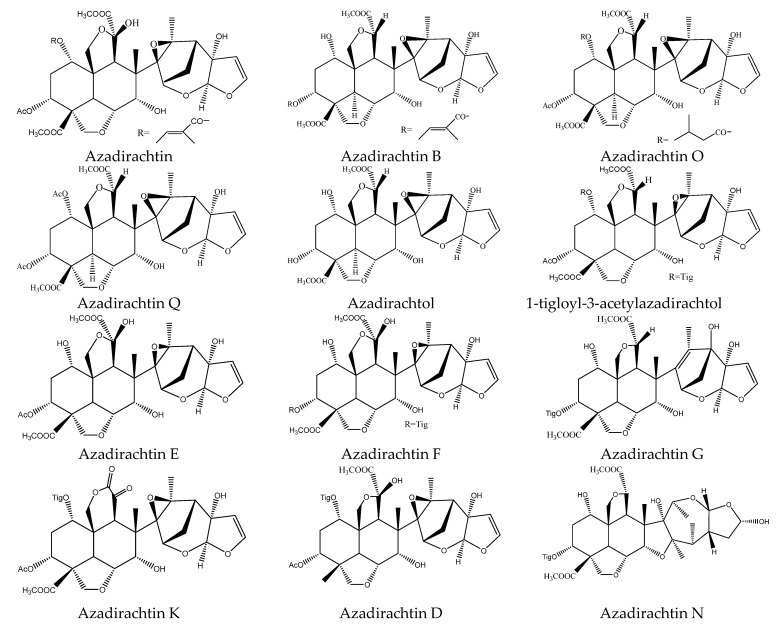
The structures of azadirachtin/meliacarpin-class chemicals.

**Figure 7:**
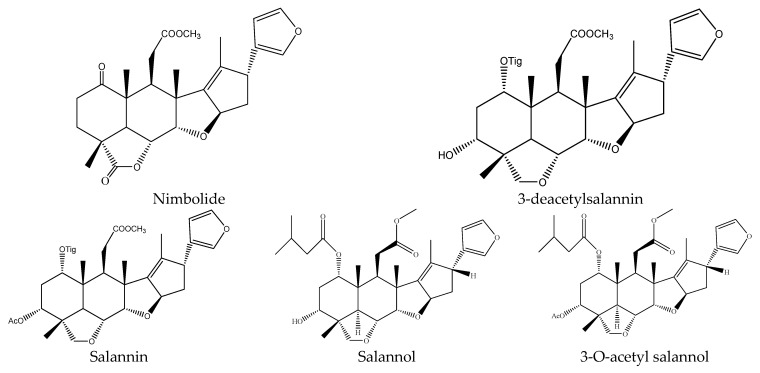
The structures of salannin-class chemicals.

**Figure 8:**
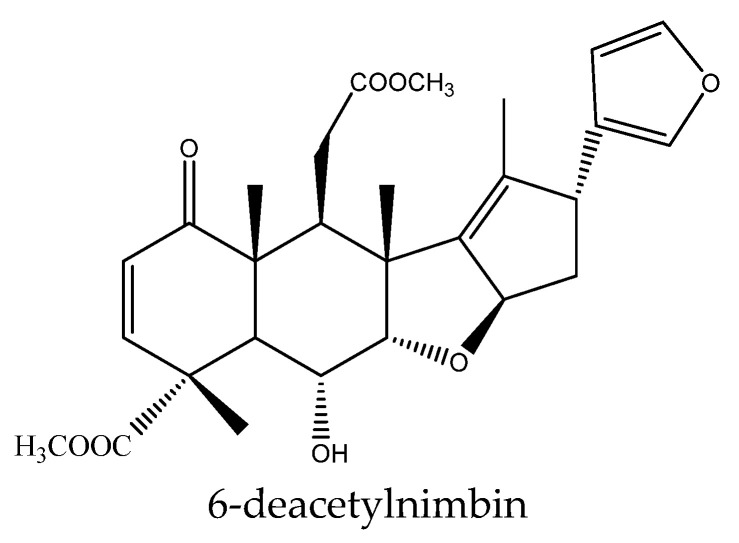
The structures of nimbin-class chemical: 6-deacetylnimbin.

**Figure 9:**
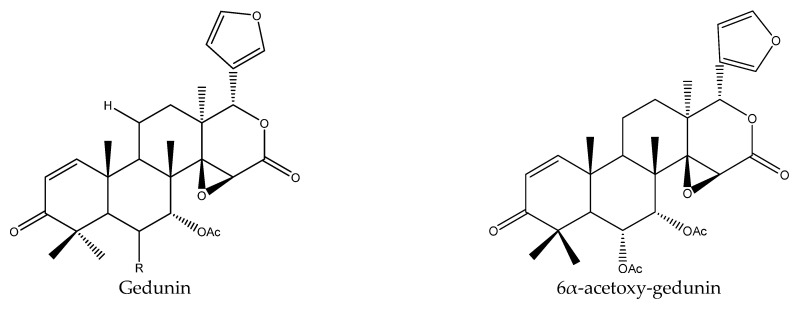

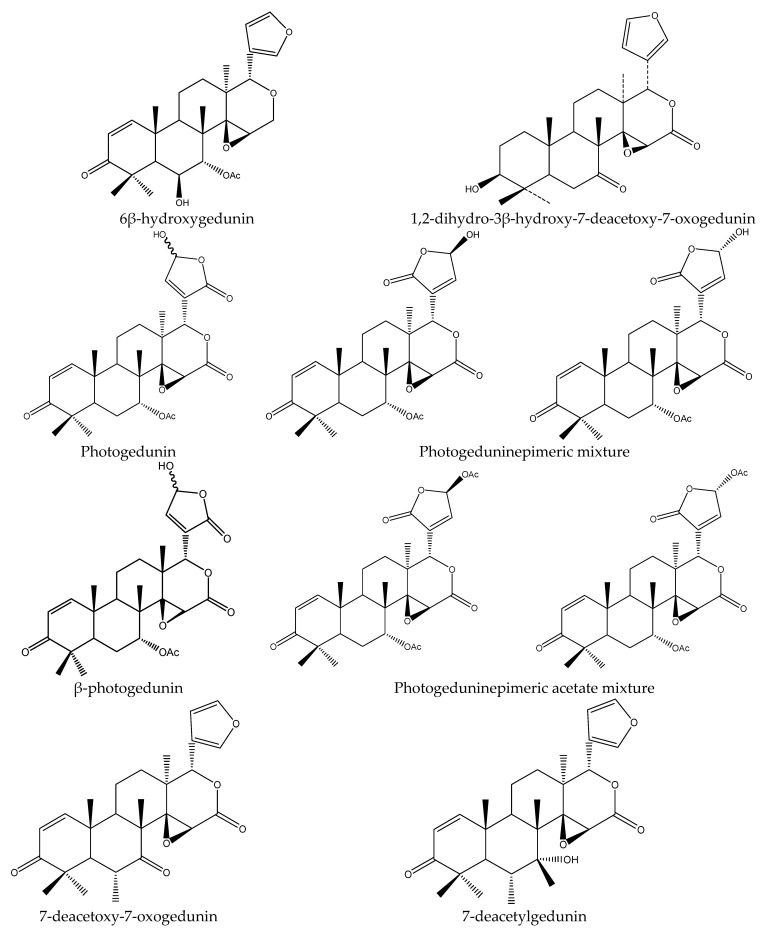
The structures of ring D-seco chemicals.

**Figure 10:**
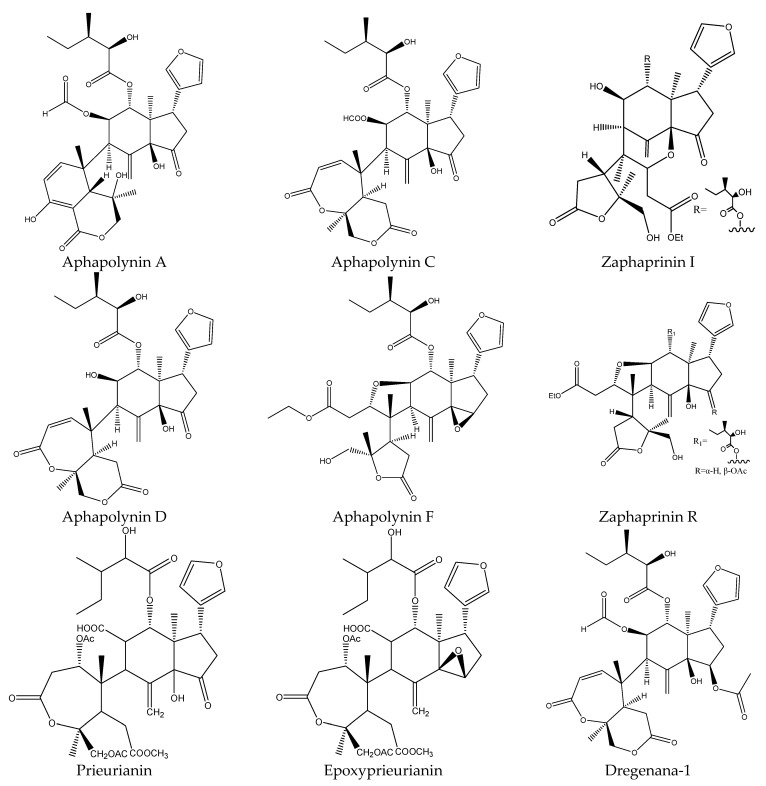
The structures of rings A,B-seco chemicals: prieurianins.

**Figure 11:**
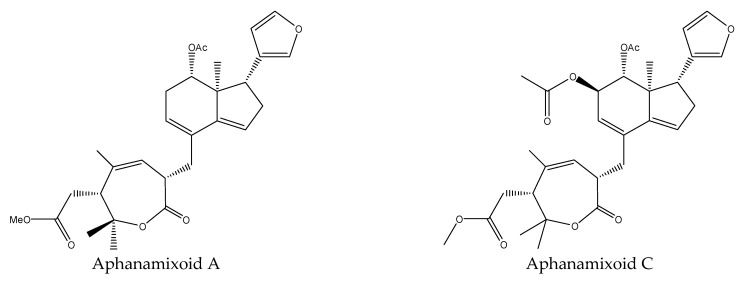

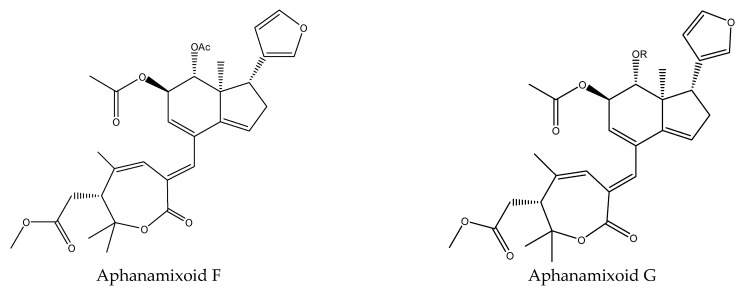
The structures of rings A,B-seco chemicals: aphanamixoids.

**Figure 12:**
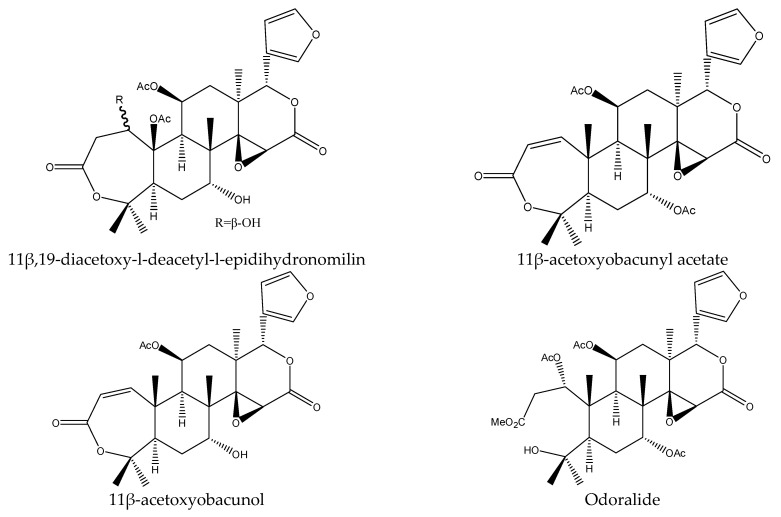
The structures of A,D-seco chemicals.

**Figure 13:**
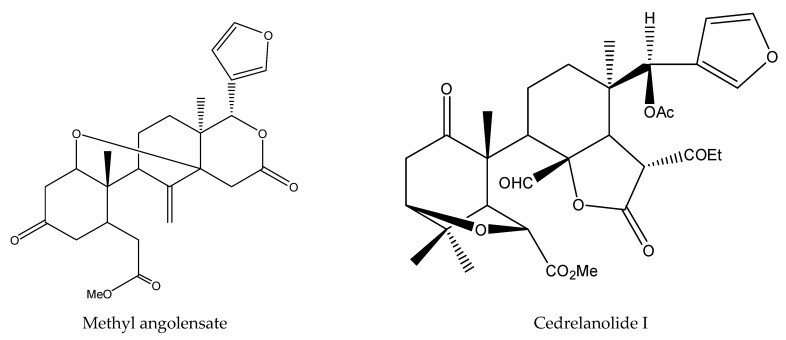
The structures of B,D-seco chemicals.

**Figure 14:**
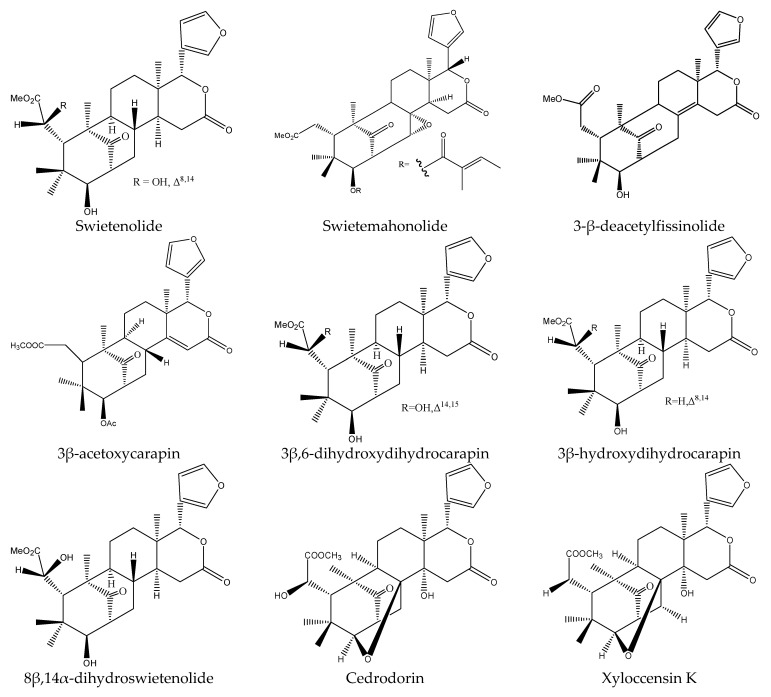

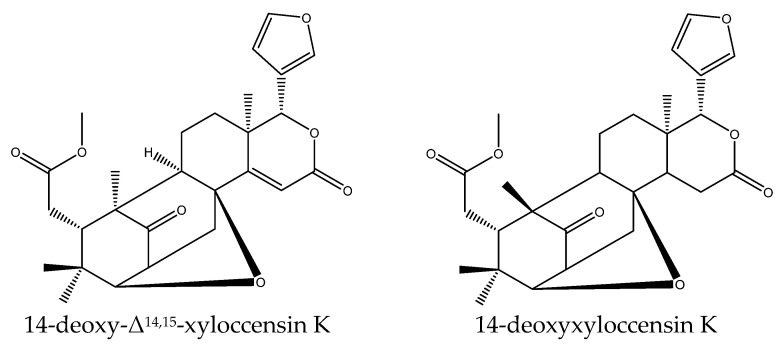
The structures of mexicanolides.

**Figure 15:**
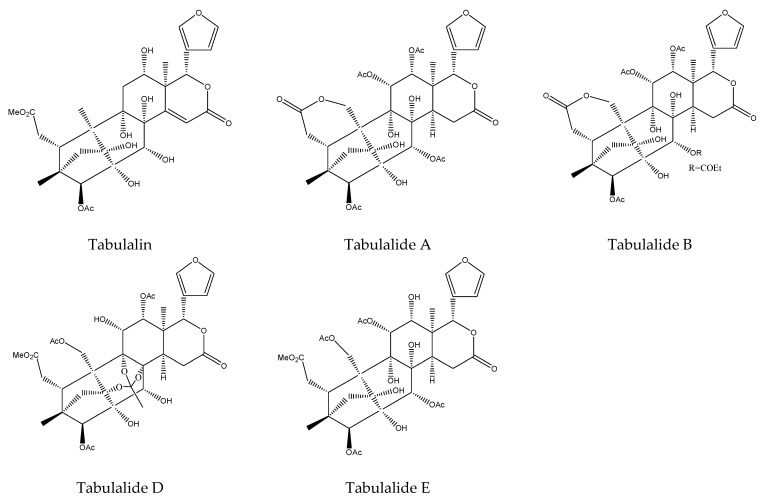
The structures of phragmalins.

**Figure 16:**
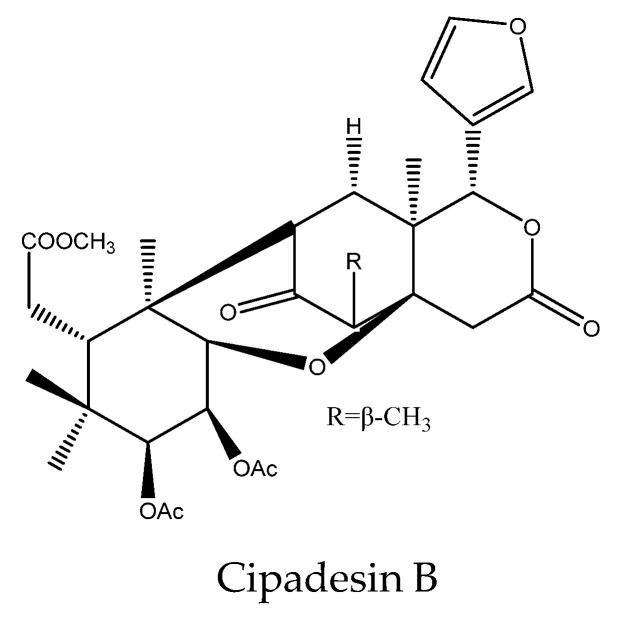
The structure of 10,11-linkage group chemical: cipadesin B.

**Figure 17:**
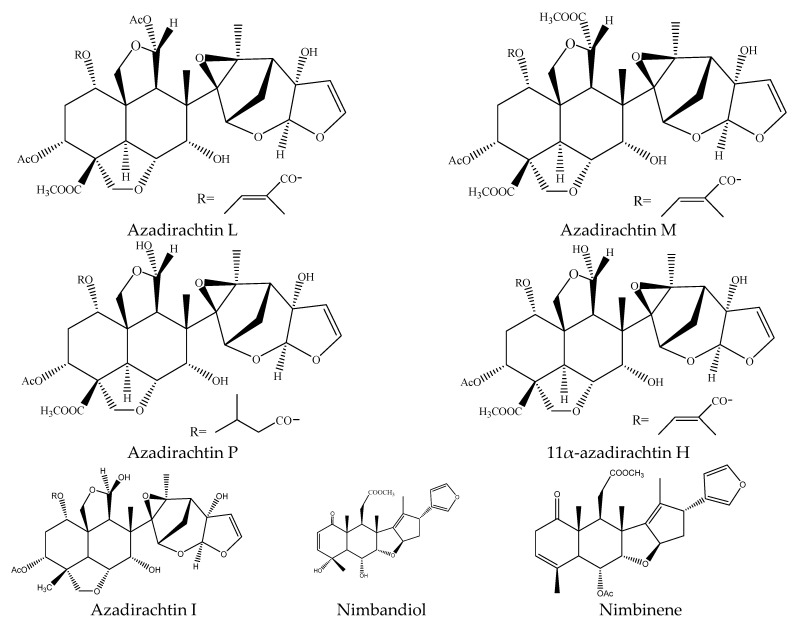
The structures of pentanortriterpenoids.

**Figure 18:**
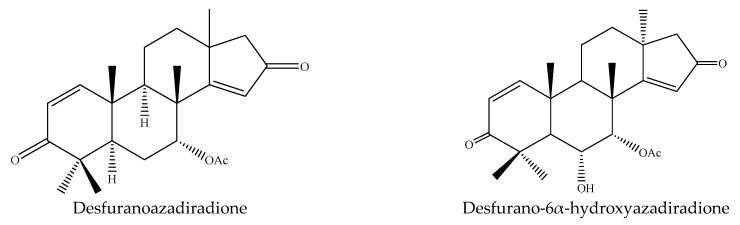
The structures of octanortriterpenoids.

**Figure 19:**
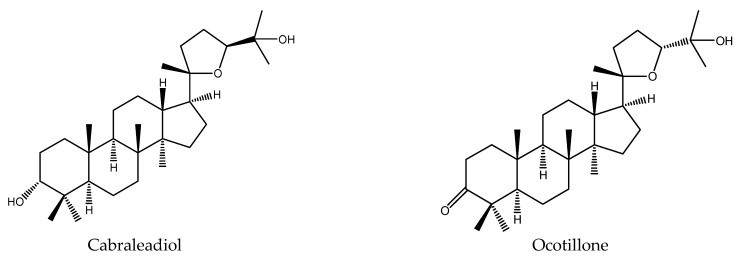
The structures of dammaranes.

**Figure 20:**
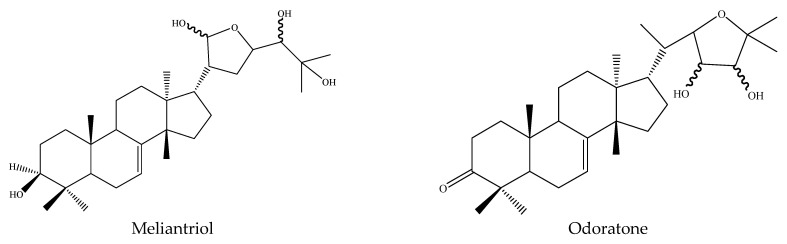
The structures of protolimonoids.

**Figure 21:**
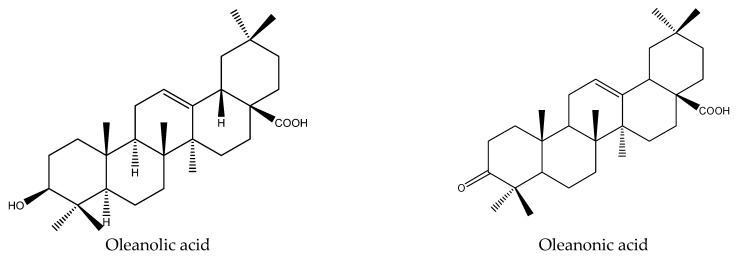
The structures of oleananes.

**Table 1:** The 19 insecticidal plant species of 8 genera in Meliaceae.

Family	Genus	Species
Meliaceae	*Aglaia*	*Aglaia elaeagnoidea* (A. Juss.) Benth.
	*Aphanamixis*	*Aphanamixis grandifolia* Bl.
		*Aphanamixis polystachya* (Wall.) R. Parker
	*Azadirachta*	*Azadirachta excelsa* (Jack) Jacobs
		*Azadirachta indica* A. Juss
		*Azadirachta siamensis* Val.
	*Cabralea*	*Cabralea canjerana* (Vell.) Mart
	*Carapa*	*Carapa guianensis* Aubl.
	*Cedrela*	*Cedrela dugessi* (S. Watson)
		*Cedrela fissilis* Vell.
		*Cedrela odorata* L.
		*Cedrela salvadorensis* L.
		*Cedrela sinensis* Juss.
		*Cedrela toona* Roxb. Ex Rottler et Willd.
		*Chisocheton ceramicus* (Miq.) C.DC.
	*Chisocheton*	*Chisocheton paniculatus* (Roxb.) Hiern
		*Chisocheton siamensis* Craib
		*Chisocheton erythrocarpus* Hiern
	*Chukrasia*	*Chukrasia tabularis* A. Juss.

**Table 2:** Antifeedant activity of insecticidal triterpenoids of plants from 8 genera in Meliaceae.

Compound	Plant Source	Insect	Activity	Ref.
Aphanamixoid A	*Aphanamixis polystachya*	*Helicoverpa armigera*	AFD *, EC_50_ = 0.015 μmol/cm^2^ (24 h)	[31]
Aphanamixoid C	*Aphanamixis polystachya*	*Helicoverpa armigera*	AFD, EC_50_ = 0.017 μmol/cm^2^ (24 h)	[18]
Aphanamixoid F	*Aphanamixis polystachya*	*Helicoverpa armigera*	AFD, EC_50_ = 0.008 μmol/cm^2^ (24 h)	
Aphanamixoid G	*Aphanamixis polystachya*	*Helicoverpa armigera*	AFD, EC_50_ = 0.012 μmol/cm^2^ (24 h)	
Prieurianin	*Aphanamixis polystachya*	*Helicoverpa armigera*	AFD, EC_50_ = 18.8 μg/mL (7 d)	[34]
Epoxyprieurianin	*Aphanamixis polystachya*	*Helicoverpa armigera*	AFD, EC_50_ = 3.2 μg/mL (7 d)	[34]
Azadirachtin	*Azadirachta indica* *Azadirachta excelsa*	*Epilachna varivesti*	AFD, EC_50_ = 13 μg/mL (24 h)	[9,10,11,12,13,15,16,33,35,36]
		*Epilachna paenulata*	AFD, LD_50_ = 1.24 μg/cm^2^ (96 h)	
		*Helicoverpa armigera*	AFD, EC_50_ = 0.26 μg/mL (6 h)	
		*Locusta migratoria*	AFD, MIC = 25 μg/mL	
		*Locusta migratoria*	AFD, ED_50_ = 3 μg/mL (48 h)	
		*Ostrinia nubilalis*	AFD, PC_50_ = 3.5 μg/mL (48 h)	
		*Peridroma saucia*	AFD, EC_50_ = 0.26 μg/mL (72 h)	
		*Pieris rapae*	AFD, AR = 100(1000 μg/mL) (24 h)	
		*Phyllotreta striolata*	AFD, MIC = 10 μg/mL	
		*Reticulitermes speratus*	AFD, PC_95_ = 65.293 (25 d)	
		*Rhodnius prolixus*	AFD, ED_50_ = 25.0 μg/mL (25 d)	
		*Schistocerca gregaria*	AFD, ED_50_ = 0.001 μg/mL	
		*Spodoptera littoralis*	AFD, AI = 98.8 ± 1.11 (1 μg/mL) (8 h)	
Azadirone	*Azadirachta indica*	*Leptinotarsa decemlineata*	AI = 11.6–26.9(100–500 μg/mL) (20 h)	[37]
7-deacetylgedunin	*Azadirachta indica*	*Reticulitermes speratus*	AFD, PC_95_ = 113.7 μg/disc (30 d)	[23]
	*Cedrela fissilis*			
	*Cedrela sinensis*			
Chisocheton compound F	*Chisocheton paniculatus*	*Pieris brassicae*	Antifeedant activity	[38]
Salannin	*Azadirachta indica*	*Reticulitermes speratus*	AFD, PC_95_ = 203.3 μg/disc (30 d)	[23]
		*Spodoptera litura*	FRA_50_^#^ = 2.8 µg/cm^2^ (7 d)	[22]
Gedunin	*Azadirachta indica*	*Reticulitermes speratus*	AFD, PC_95_ = 218.4 μg/disc (30 d)	[23]
	*Cedrela dugessi*			
	*Cedrela fissilis*			
	*Cedrela sinensis*			
	*Cedrela salvadorensis*			
	*Cabralea eichleriana*			
	*Carapa guianensis*			
	*Chisocheton paniculatus*			
17β-hydroxy- azadiradione	*Azadirachta indica*	*Reticulitermes speratus*	AFD, PC_95_ = 235.6 μg/disc (30 d)	[23]
	*Carapa guianensis*			
nimbandiol	*Azadirachta indica*	*Reticulitermes speratus*	AFD, PC_95_ = 254.4 μg/disc (30 d)	[23]
3-deacetylsalannin	*Azadirachta indica*	*Reticulitermes speratus*	AFD, PC_95_ = 1373.1 μg/disc (30 d)	[23]
6-deacetylnimbin	*Azadirachta indica*	*Reticulitermes speratus*	AFD, PC_95_ = 1581.2 μg/disc (30 d)	[23]
Azadirachtin B	*Azadirachta indica*	*Locusta migratoria*	AFD, EC_50_ = 12 μg/mL	[39]
	*Azadirachta excelsa*	*Epilachna varivesti*	AFD, EC_50_ = 30 μg/mL	[9]
Nimbolide	*Azadirachta indica*	*Epilachna varivesti*	AFD, EC_50_ = 90 μg/mL	[9]
	*Azadirachta excelsa*			
Azadirachtin L	*Azadirachta indica*	*Epilachna varivesti*	AFD, EC_50_ = 6 μg/mL	[9]
	*Azadirachta excelsa*			
1-tigloyl-3-acetyl- azadirachtol	*Azadirachta excelsa*	*Epilachna varivesti*	AFD, EC_50_ = 6 μg/mL	[9]
	*Azadirachta siamensis*			
Salannol	*Azadirachta indica*	*Spodoptera litura*	FRA_50_ = 2.3 µg/cm^2^ (7 d)	[22]
Azadiraindin A	*Azadirachta indica*	*Plutella xylostella*	AR = 28% at 2000 μg/mL (48 h)	[24]
Epoxyazadiradione	*Azadirachta indica*	*Plutella xylostella*	AR = 37.2% at 2000 μg/mL (48 h)	[24]
Desfuranoazadiradione	*Azadirachta indica*	*Plutella xylostella*	AR = 39.6% at 2000 μg/mL (48 h)	[24]
Azadiradione	*Azadirachta indica*	*Plutella xylostella*	AR = 90.6% at 2000 μg/mL (48 h)	[24]
	*Chisocheton siamensis*			
7-deacetoxy-7-oxo- gedunin	*Cedrela fissilis*	*Spodoptera littoralis*	AFD at 1000 μg/mL (3–10 h)	[20]
	*Cabralea eichleriana*			
	*Carapa guianensis*			
Methyl angolensate	*Cedrela fissilis*	*Spodoptera litura*	AFD, PFI = 65.3 at 1 μg/cm^2^ (24 h)	[40]
	*Cabralea canjerana*			
11β-acetoxyobacunyl acetate	*Cedrela odorata*	*Spodoptera littoralis*	AFD at 1000 μg/mL	[29]
11β,19-diacetoxy-l-de- acetyl-l-epidihy- dronomilin	*Cedrela odorata*	*Spodoptera littoralis*	AFD at 1000 μg/mL	[29]
11β-acetoxyobacunol	*Cedrela odorata*	*Spodoptera littoralis*	AFD at 1000 μg/mL	[29]
Odoralide	*Cedrela odorata*	*Spodoptera littoralis*	AFD at 1000 μg/mL	[29]
Swietenolide	*Cedrela odorata*	*Spodoptera littoralis*	AFD at 1000 μg/mL	[29]
8β,14α-dihydro- swietenolide	*Cedrela odorata*	*Spodoptera littoralis*	AFD at 500 μg/mL	[29]
3β,6-dihydroxydihydro -carapin	*Cedrela odorata*	*Spodoptera littoralis*	AFD at 1000 μg/mL	[29]
3β-hydroxydihydro- carapin	*Cedrela odorata*	*Spodoptera littoralis*	AFD at 1000 μg/mL	[29]
Xyloccensin K	*Cedrela odorata*	*Spodoptera littoralis*	AFD at 1000 μg/mL	[29]
Cedrodorin	*Cedrela odorata*	*Spodoptera littoralis*	AFD at 1000 μg/mL	[29]
Ocotillone	*Cabralea canjerana*	*Spodoptera litura*	AFD, PFI = 44.5 at 1 μg/cm^2^ (24)	[41]
Tabulalin	*Chukrasia tabularis*	*Spodoptera littoralis*	AFD at 500 μg/mL (2–12 h)	[42]
Tabulalide D	*Chukrasia tabularis*	*Spodoptera littoralis*	AFD at 500 μg/mL (2–12 h)	[42]
TabulalideA	*Chukrasia tabularis*	*Spodoptera littoralis*	AFD at 1000 μg/mL (2–12 h)	[42]
Tabulalide B	*Chukrasia tabularis*	*Spodoptera littoralis*	AFD at 1000 μg/mL (2–12 h)	[42]
Tabulalide E	*Chukrasia tabularis*	*Spodoptera littoralis*	AFD at 1000 μg/mL (2–12 h)	[42]

*: AFD means antifeedant activity; ^#^: FRA_50_ means feeding reducing activity by 50%.

**Table 3:** Poisonous activity of insecticidal triterpenoids of plants from 8 genera in Meliaceae.

Compound	Plant Source	Insect	Activity	Ref.
Aphapolynin D	*Aphanamixis polystachya*	*Diabrotica balteata*	MS: 66 5–9 d)	[19]
Aphanalide F	*Aphanamixis polystachya*	*Diabrotica balteata*	MS: 66 (5–9 d)	
Aphapolynin F	*Aphanamixis polystachya*	*Diabrotica balteata*	MS: 33 (5–9 d)	
Dregenana-1	*Aphanamixis polystachya*	*Diabrotica balteata*	MS: 33 (5–9 d)	
Aphanalide E	*Aphanamixis polystachya*	*Diabrotica balteata*	MS: 33 (5–9 d)	
Aphanalide G	*Aphanamixis polystachya*	*Diabrotica balteata*	MS: 33 (5–9 d)	
Aphanalide H	*Aphanamixis polystachya*	*Diabrotica balteata*	MS: 99 (5–9 d)	
Aphapolynin C	*Aphanamixis polystachya*	*Diabrotica balteata*	MS: 99 (5–9 d)	
	*Aphanamixis polystachya*	*Caenorhabditis elegans*	MS: 66 (5–9 d)	
Aphapolynin A	*Aphanamixis polystachya*	*Plutella xylostella*	MS: 66 (5–9 d)	
Zaphaprinin I	*Aphanamixis polystachya*	*Plutella xylostella*	MS: 99 (5–9 d)	
Zaphaprinin R	*Aphanamixis polystachya*	*Plutella xylostella*	MS: 99 (5–9 d)	
Azadirachtin	*Azadirachta indica Azadirachta excelsa*	*Spodoptera littoralis*	LC_50_ = 0.32 μg/mL (12 d)	[9,10,11,12,13,15,16,33,35,36]
		*Anopheles gambiae*	LD_50_ = 57.1 μg/mL (24 h)	
		*Plutella xylostella*	LD_50_ = 7.04–0.87 (24–96 h)	
7-deacetylgedunin	*Azadirachta indica*	*Atta sexdens rubropilosa*	S_50_ = 9 d at 100 μg/mL	[28]
	*Cedrela fissilis*			
	*Cedrela sinensis*			
Gedunin	*Azadirachta indica*	*Spodoptera frugiperda*	LC_50_ = 39 μg/mL (7 d)	[43]
	*Cedrela dugessi*			
	*Cedrela fissilis*			
	*Cedrela sinensis*			
	*Cedrela salvadorensis*			
	*Cabralea eichleriana*			
	*Carapa guianensis*			
	*Chisocheton paniculatus*			
Nimocinol	*Azadirachta indica*	*Aedes aegypti*	LC_50_ = 21 μg/mL (24 h)	[25]
6α-O-acetyl-7-deacetyl- nimocinol	*Azadirachta indica*	*Aedes aegypti*	LC_50_ = 83 μg/mL (24 h)	[25]
22,23-dihydronimocinol	*Azadirachta indica*	*Anopheles stephensi*	LC_50_ = 60 μg/mL (24 h)	[26]
desfurano-6α-hydroxy- azadiradione	*Azadirachta indica*	*Anopheles stephensi*	LC_50_ = 43 μg/mL (24 h)	[26]
Meliatetraolenone	*Azadirachta indica*	*Anopheles stephensi*	LC_50_ = 16 μg/mL (24 h)	[26]
Odoratone	*Azadirachta indica*	*Anopheles stephensi*	LC_50_ = 154 μg/mL (24 h)	[44]
Azadirachtin O	*Azadirachta excelsa*	*Plutella xylostella*	LD_50_ = 3.92 μg/g (24 h)	[33]
Azadirachtin P	*Azadirachta excelsa*	*Plutella xylostella*	LD_50_ = 2.19 μg/g (24 h)	[33]
Azadirachtin Q	*Azadirachta excelsa*	*Plutella xylostella*	LD_50_ = 1.10 μg/g (96 h)	[33]
Azadirachtin B	*Azadirachta excelsa*	*Plutella xylostella*	LD_50_ = 1.06 μg/g (96 h)	[33]
Azadirachtin L	*Azadirachta excelsa*	*Plutella xylostella*	LD_50_ = 1.92 μg/g (96 h)	[33]
Azadirachtin M	*Azadirachta excelsa*	*Plutella xylostella*	LD_50_ = 1.30 μg/g (96 h)	[33]
11α-azadirachtin H	*Azadirachta excelsa*	*Plutella xylostella*	LD_50_ = 0.75 μg/g (96 h)	[33]
Azadirachtol	*Azadirachta excelsa*	*Plutella xylostella*	LD_50_ = 1.78 μg/g (96 h)	[33]
23-O-methylnimocinolide	*Azadirachta indica*	*Aedes aegypti*	LC_50_ = 53 μg/mL (24 h)	[45]
7-O-deacetyl-23-O-methyl- 7α-O-senecioyl-nimocinolide	*Azadirachta indica*	*Aedes aegypti*	LC_50_ = 14 μg/mL (24 h)	[45]
6α-acetoxygedunin	*Aglaia elaeagnoidea*	*Atta sexdens rubropilosa*	S_50_ = 8 d at 100 μg/mL	[28]
	*Carapa guianensis*			
	*Cedrela fissilis*			
	*Chisocheton* *paniculatus*			
14-deoxy-Δ^14,15^-xyloccensin K	*Chisocheton erythrocarpus Hiern*	*Aedes aegypti,* *Aedes albopictus* *Culex Quinquefasciatus*	LC_50_ = 10.2 μg/mL (24 h) LC_50_ = 12.16 μg/mL (24 h) LC_50_ = 16.82 μg/mL (24 h)	[46]
14-deoxyxyloccensin K	*Chisocheton erythrocarpus Hiern* *Chisocheton ceramicus*	*Aedes aegypti,* *Aedes albopictus* *Culex Quinquefasciatus*	LC_50_ = 3.19 μg/mL (24 h) LC_50_ = 3.01 μg/mL (24 h) LC_50_ = 3.64 μg/mL (24 h)	[46]
Photogedunin epimer mixture	*Cedrela dugessi*	*Spodoptera frugiperda*	LC_50_ = 10 μg/mL (7 d)	[47]
Photoacetic acid acetate mixture	*Cedrela dugessi*	*Spodoptera frugiperda*	LC_50_ = 8 μg/mL (7 d)	[47]
7-deacetoxy-7-oxo-gedunin	*Cedrela fissilis*	*Atta sexdens rubropilosa*	S_50_ = 11 d at 100 μg/mL	[28]
	*Cabralea eichleriana*			
	*Carapa guianensis*			
Photogedunin	*Cedrela fissilis*	*Atta sexdens rubropilosa*	S_50_ = 9 d at 100 μg/mL	[28]
1,2-dihydro-3β-hydroxy-7- deacetoxy-7-oxogedunin	*Cedrela fissilis*	*Atta sexdens rubropilosa*	S_50_ = 9 d at 100 μg/mL	
Cipadesin B	*Cedrela fissilis*	*Atta sexdens rubropilosa*	S_50_ = 9 d at 100 μg/mL	[28]
Swietemahonolide	*Cedrela fissilis*	*Atta sexdens rubropilosa*	S_50_ = 8 d at 100 μg/mL	
3β-acetoxycarapin	*Cedrela fissilis*	*Atta sexdens rubropilosa*	S_50_ = 8 d at 100 μg/mL	
Oleanolic acid	*Cedrela fissilis*	*Atta sexdens rubropilosa*	S_50_ = 6 d at 100 μg/mL	
Oleanonic acid	*Cedrela fissilis*	*Atta sexdens rubropilosa*	S_50_ = 8 d at 100 μg/mL	
Methyl angolensate	*Cedrela fissilis*	*Spodoptera frugiperda*	MR: 40% at 50 mg/kg (7 d)	[48]
	*Cabralea canjerana*			
Photogeduninepimeric acetate mixture	*Cedrela salvadorensis*	*Spodoptera frugiperda*	SR 50% at 10 μg/mL (24 h)	[49]
Photogeduninepimeric mixture	*Cedrela salvadorensis*	*Spodoptera frugiperda*	SR 17% at 10 μg/mL (24 h)	
Ocotillone	*Cabralea canjerana*	*Spodoptera frugiperda*	MR: 40% at 50 mg/kg (7 d)	[48]
β-photogedunin	*Carapa guianensis*	*Spodoptera frugiperda*	LM 53.3% at 50 μg/mL (7 d)	[48]
			PM 20.0% at 50 μg/mL (7 d)	

MS: mortality scored; SR: survival rate; MR: mortality rate; LM: larval mortality; PM: pupal mortality.

**Table 4:** Growth regulatory activity of insecticidal triterpenoids of plants from 8 genera in Meliaceae.

Compound	Plant Source	Insect	Activity	Ref.
Azadirachtin	*Azadirachta indica* *Azadirachta excelsa*	*Helicoverpa armigera*	IGR, EC_50_ = 0.26 μg/mL (7 d)	[9,10,11,12,13,15,16,33,35,36]
		*Rhodnius prolixus*	IGR, ED_50_ = 0.40 μg/mL (7 d)	
		*Heliothis zea* *Heliothis virescens*	IGR, ED_50_ = 0.70 μg/mL (10 d)	
		*Spodoptera frugiperda,* *Pectinophora gossypiella*	IGR, ED_50_ = 0.40 μg/mL (10 d)	
		*Spodoptera litura*	IGR, EC_50_ = 0.21 μg/mL (7 d)	
		*Spodoptera littoralis*	EC_50_ = 0.11 μg/mL (6 d)	
Nimocinolide	*Azadirachta indica*	*Musca domestica*	FI at 100 μg/mL	[27]
Isonimocinolide	*Azadirachta indica*	*Musca domestica*	FI at 100 μg/mL	[27]
		*Aedes uegypti*	mutagenic properties	
7-deacetylazadiradione	*Azadirachta indica*	*Heliothis virescens*	IGR, EC_50_ = 1600 μg/mL	[30]
	*Chisocheton paniculatus*			
Salannin	*Azadirachta indica*	*Helicoverpa armigera*	IGR EC_50_ = 86.5 μg/mL (7 d)	[22]
	*Azadirachta indica*	*Spodoptera litura*	IGR EC_50_ = 87.7 μg/mL (7 d)	
3-O-acetyl salannol	*Azadirachta indica*	*Helicoverpa armigera*	IGR EC_50_ = 64.2 μg/mL (7 d)	[22]
	*Azadirachta indica*	*Spodoptera litura*	IGR EC_50_ = 65.6 μg/mL; RF_50_ at 2.0 µg/cm^2^ (7 d)	
Salannol	*Azadirachta indica*	*Helicoverpa armigera*	IGR, EC_50_ was 79.7 μg/mL (7 d)	[22]
	*Azadirachta indica*	*Spodoptera litura*	IGR, EC_50_ = 77.4 μg/mL (7 d)	
6β-hydroxygedunin	*Azadirachta indica*	*Helicoverpa armigera*	IGR EC_50_ = 24.2 μg/mL (7 d)	[35]
	*Azadirachta indica*	*Spodoptera litura*	IGR EC_50_= 391.4 μg/mL (7 d)	
Nimbinene	*Azadirachta indica*	*Helicoverpa armigera*	IGR EC_50_ was 21.5 μg/mL (7 d)	[35]
	*Azadirachta indica*	*Spodoptera litura*	IGR EC_50_ = 404.5 μg/mL (7 d)	
Azadiradione	*Azadirachta indica*	*Heliothis virescens*	IGR, EC_50_= 560 μg/mL	[30]
	*Chisocheton siamensis*			
	*Azadirachta indica*	*Heliothis virescens*	IGR, EC_50_ = 560 μg/mL	[30]
	*Chisocheton siamensis*			
6α-acetoxygedunin	*Aglaia elaeagnoidea*	*Ostrinia nubilalis*	reduced growth at 50 μg/mL	[17]
	*Carapa guianensis*			
	*Cedrela fissilis*			
	*Chisocheton paniculatus*			
Cedrelanolide I	*Cedrela salvadorensis*	*Ostrinia nubilalis*	reduced weight at 50 μg/mL	[51]
Cedrelone	*Cedrela odorata*	*Peridroma saucia*	IGR, EC_50_ = 53.1 μg/mL (9 d)	[29]
	*Cedrela toona*			
Cabraleadiol	*Cabralea canjerana*	*Spodoptera frugiperda*	LPE, 1.2 d	[48]
3β-deacetylfissinolide	*Cabralea canjerana*	*Spodoptera frugiperda*	LPE, 1.2 d	[48]
β-photogedunin	*Carapa guianensis*	*Spodoptera frugiperda*	PWI at 50 mg/kg (7 d)	[48]
Cedrelanolide I	*Cedrela salvadorensis*	*Ostrinia nubilalis*	reduced weight at 50 μg/mL	[51]
Meliantriol	*Azadirachta indica*	*Locusts*	chewing prevention	[52]
7-deacetyl-17β-hydroxy-azadiradione	*Azadirachta indica*	*Heliothis virescens*	IGR, EC_50_ = 240 μg/mL	[30]

IGR: insect growth inhibitory activity; LPE: larval phase extended; FI: fecundity inhibition; RF_50_: reduced feeding by 50%; PWI: pupal weight inhibition.
